# Burden of and Trends in Urticaria Globally, Regionally, and Nationally from 1990 to 2019: Systematic Analysis

**DOI:** 10.2196/50114

**Published:** 2023-10-26

**Authors:** Xiaoli Liu, Yuan Cao, Wenhui Wang

**Affiliations:** 1 Research Center of Clinical Epidemiology Peking University Third Hospital Beijing China; 2 Department of Hernia and Abdominal Wall Surgery Beijing Chaoyang Hospital Capital Medical University Beijing China; 3 Department of Dermatology Peking University Third Hospital Beijing China

**Keywords:** urticaria, burden of disease, prevalence, incidence, disability-adjusted life years

## Abstract

**Background:**

Urticaria presents a significant global health challenge due to its sudden onset and potential for severe allergic reactions. Past data on worldwide prevalence and incidence is inconsistent due to differing study methodologies, regional differences, and evolving diagnostic criteria. Past studies have often provided broad ranges instead of specific figures, underscoring the necessity for a cohesive global perspective to inform public health strategies.

**Objective:**

We aimed to assess the global burden of urticaria using the 2019 Global Burden of Disease (GBD) study data and systematically analyze urticaria prevalence, incidence, and disability-adjusted life years (DALYs) at global, regional, and national levels, thereby informing more effective prevention and treatment strategies.

**Methods:**

We analyzed the global, regional, and national burden of urticaria from 1990 to 2019 using the 2019 GBD study coordinated by the Institute for Health Metrics and Evaluation. Estimations of urticaria prevalence, incidence, and DALYs were derived using DisMod-MR 2.1, a Bayesian meta-regression tool. The Socio-demographic Index (SDI) was used to correlate development status with health outcomes. The GBD’s division of the world into 21 regions and 204 countries and territories facilitated a comprehensive assessment. Age-standardized estimated annual percentage changes were generated for urticaria metrics to quantify temporal trends, with age standardization adjusting for potential confounding from age structure.

**Results:**

From 1990 to 2019, the global age-standardized prevalence, incidence, and DALY rates of urticaria showed marginal changes. In 2019, 65.14 million individuals were affected, with a prevalence rate of 841.88 per 100,000 population. The DALY rate was 50.39 per 100,000 population. Compared to 1990, the global age-standardized prevalence, incidence, and DALY rates saw increases of 2.92, 4.84, and 0.31 per 100,000 population, respectively. Women persistently had higher rates than men. At a regional level in 2019, low-middle SDI regions exhibited the highest age-standardized metrics, whereas high SDI regions reported the lowest. Central Europe showed the highest rates, contrasting with Western Europe’s lowest rates. Nationally, urticaria prevalence in 2019 varied dramatically, from a low of 27.1 per 100,000 population in Portugal to a high of 92.0 per 100,000 population in Nepal. India reported the most DALYs at 749,495.9, followed by China, Pakistan, and the United States. Agewise data showed higher rates in younger age groups, which diminished with age and then experienced a slight resurgence in the oldest populations. This pattern was pronounced in women and younger populations, with the largest rises seen in those aged less than 40 years and the smallest in those aged more than 70 years.

**Conclusions:**

Urticaria remains a significant global health issue, with considerable variation across regions, countries, and territories. The increased burden among women, the rising burden in younger populations, and the regional differences in disease burden call for tailored interventions and policies to tackle this emerging public health issue.

## Introduction

Urticaria, commonly known as hives, is a dermatologic condition characterized by the sudden onset of itchy, red, and raised welts or bumps on the skin [1,2]. These hives can appear anywhere on the body and vary in size and shape, often causing discomfort and distress to patients [2,3]. In addition to the physical symptoms, urticaria can also have a significant impact on patients’ quality of life, affecting their social and psychological well-being [4,5]. The global burden of urticaria has been a growing concern in recent years, with its high prevalence and potential to cause severe allergic reactions and even anaphylaxis in some cases [1,6]. Understanding the global epidemiology of urticaria is crucial for improving prevention and treatment strategies and reducing its impact on public health [7-9].

Despite the high prevalence of urticaria, there is limited information on its global burden [10]. A systematic review and meta-analysis of 52 studies from different regions of the world found that the prevalence of chronic urticaria ranged from 0.1% to 8.9%, with a median of 0.8% [11]. The variation in reported prevalence could be due to differences in study design, sample size, and diagnostic criteria. Furthermore, the burden of urticaria in terms of incidence has not been well documented. A study from the United States estimated that the annual incidence of acute urticaria was 119 per 100,000 population, while the incidence of chronic urticaria was 20 per 100,000 population [12]. Another study from Germany reported a higher incidence of chronic urticaria: 0.15% among a total of 3.53 million individuals [9]. However, these estimates may not be generalizable to other regions of the world.

Understanding the burden of urticaria is crucial for guiding the allocation of health care resources and developing effective prevention and treatment strategies. For instance, a study from Brazil found that adults with chronic urticaria have substantially worse outcomes than people living without chronic urticaria, including health-related quality of life decrements, anxiety, and sleep difficulties [13]. Chronic urticaria was also associated with significant impairments in work and nonwork activities and greatly elevated health care resource use [13]. Another study found that the quality of life of patients with chronic urticaria was significantly lower than that of the general population, underscoring the impact of urticaria on patients’ well-being [4].

More research is needed to understand the epidemiology and burden of urticaria worldwide. The Global Burden of Disease (GBD) study aims to provide a systematic analysis of the burden of various human diseases, including urticaria, from 1990 to 2019, offering insights into their prevalence, incidence, disability-adjusted life years (DALYs), and trends. The study can identify areas with the highest burden and inform public health policies and resource allocation. Our study’s purpose is to assess the global burden of urticaria using the 2019 GBD study data, examining prevalence, incidence, and DALYs at global, regional, and national levels. The significance lies in informing prevention and treatment strategies, reducing urticaria’s impact on human health, and improving quality of life worldwide.

## Methods

### Data Sources and Study Design

The 2019 GBD study was used to analyze the global, regional, and country urticaria burden from 1990 to 2019. The Institute for Health Metrics and Evaluation (IHME) coordinates the GBD study, which assesses illness, injury, and risk factor burden in 204 nations and territories. The GBD Results tool, an online data repository, provided the latest GBD estimations for our investigation.

To estimate the prevalence, incidence, and DALYs of urticaria, we used DisMod-MR 2.1, a Bayesian meta-regression tool developed by the IHME for modeling disease epidemiology. This tool allows for the integration of various data sources, including surveys, administrative records, and published studies, while accounting for potential biases and heterogeneity in the data.

### Socio-Demographic Index

The Socio-demographic Index (SDI) is a composite indicator expressed on a scale of 0 to 1 of development status that is strongly correlated with health outcomes. It is the geometric mean of indices of lag-distributed income per capita, mean years of schooling for individuals aged 15 years or more, and total fertility rate for individuals aged less than 25 years. A location with an SDI of 0 indicates a theoretical minimum level of development status relevant to health outcomes, while a location with an SDI of 1 indicates a theoretical maximum level.

The SDI is stratified into quintiles to provide nuanced distinctions in sociodemographic development. Specifically, a low SDI spans from 0 to 0.454743, followed by a low-middle SDI ranging from 0.454743 to 0.607679. The middle SDI encompasses values between 0.607679 and 0.689504. Subsequently, the high-middle SDI covers the range of 0.689504 to 0.805129, and finally, a high SDI is defined from values of 0.805129 up to 1.

### Regions, Countries, and Territories

The GBD study divides the world into 21 regions based on geographical location and epidemiological similarity. These regions are further subdivided into 204 countries and territories. In our analysis, we assessed the burden of urticaria for each region and country, enabling us to identify areas with the highest disease burden and potential targets for intervention.

### Statistical Analysis

Urticaria prevalence, incidence, and DALYs were computed for each SDI, area, and nation. We generated age-standardized estimated annual percentage changes (EAPCs) to quantify urticaria incidence, prevalence, and DALYs. Age-standardized incidence, prevalence, and DALY rates of urticaria were compared across populations after they were adjusted for potential age structure confounding by applying the age-specific rates for each location, gender, and year to a GBD world standard population.

The EAPC is a widely used measure of the age-standardized rate trend over a specified time interval [14]. We fitted a regression line to the natural logarithm of the age-standardized rate to calculate the EAPC: y = α + βx + ε, where y = ln (age-standardized rate) and x = calendar year. This was then expressed as a percentage: 100 × (eβ − 1). The 95% CI of the EAPC was calculated to reflect the temporal trend in the age-standardized rate. An upward trend in the age-standardized rate was indicated when the EAPC and the lower boundary of the 95% CI were positive, while a downward trend was indicated when the EAPC and the upper boundary of the 95% CI were negative. We calculated the EAPCs for age-standardized incidence rates, age-standardized prevalence rates, and age-standardized DALY rates of urticaria to reflect their temporal trends. All data analyses were conducted using R (version 4.2.1; R Foundation for Statistical Computing) and Origin (2022 version; OriginLab).

### Ethical Considerations

Our research is based on the secondary analysis of data derived from the 2019 GBD study. This comprehensive study integrated data from a diverse array of primary sources, including various surveys, censuses, vital statistics, and other health-related data sets that previously received ethical approval from their respective institutional review boards. Given that our study leverages secondary data, which is an aggregation and anonymization of these primary data sets, our analysis did not necessitate a separate institutional review board approval or exemption [14].

## Results

### Global Level

**Figure 1:**
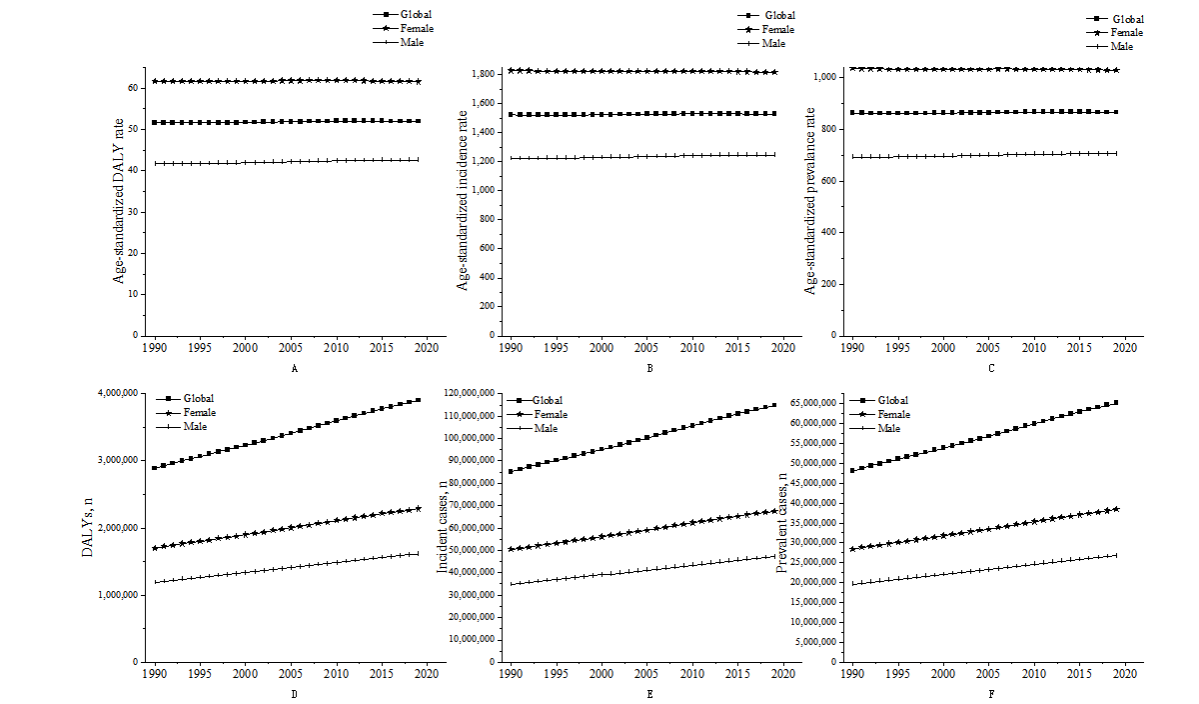
Global, female, and male age-standardized urticaria DALY rate (A), incidence rate (B), prevalence rate (C), DALY number (D), incident cases (E), and prevalent cases (F) from 1990 to 2019. DALY: disability-adjusted life year.

From 1990 to 2019, the global age-standardized prevalence, incidence, and DALY rates of urticaria remained stable, with women having higher rates than men. The number of cases, DALYs, and incidence increased during this period. In 2019, 65.14 million people had urticaria, with a prevalence of 841.88 per 100,000 population and an age-standardized prevalence rate of 865.51 per 100,000 population. The global DALY rate was 50.39 per 100,000 population, with an age-standardized rate of 51.91 per 100,000 population. Compared to 1990, the global age-standardized prevalence, incidence, and DALY rates increased by 2.92, 4.84, and 0.31 per 100,000 population, respectively, in 2019 (Figure 1; Multimedia Appendix 1, Tables S1 and S2).

### Regional Level

**Figure 2:**
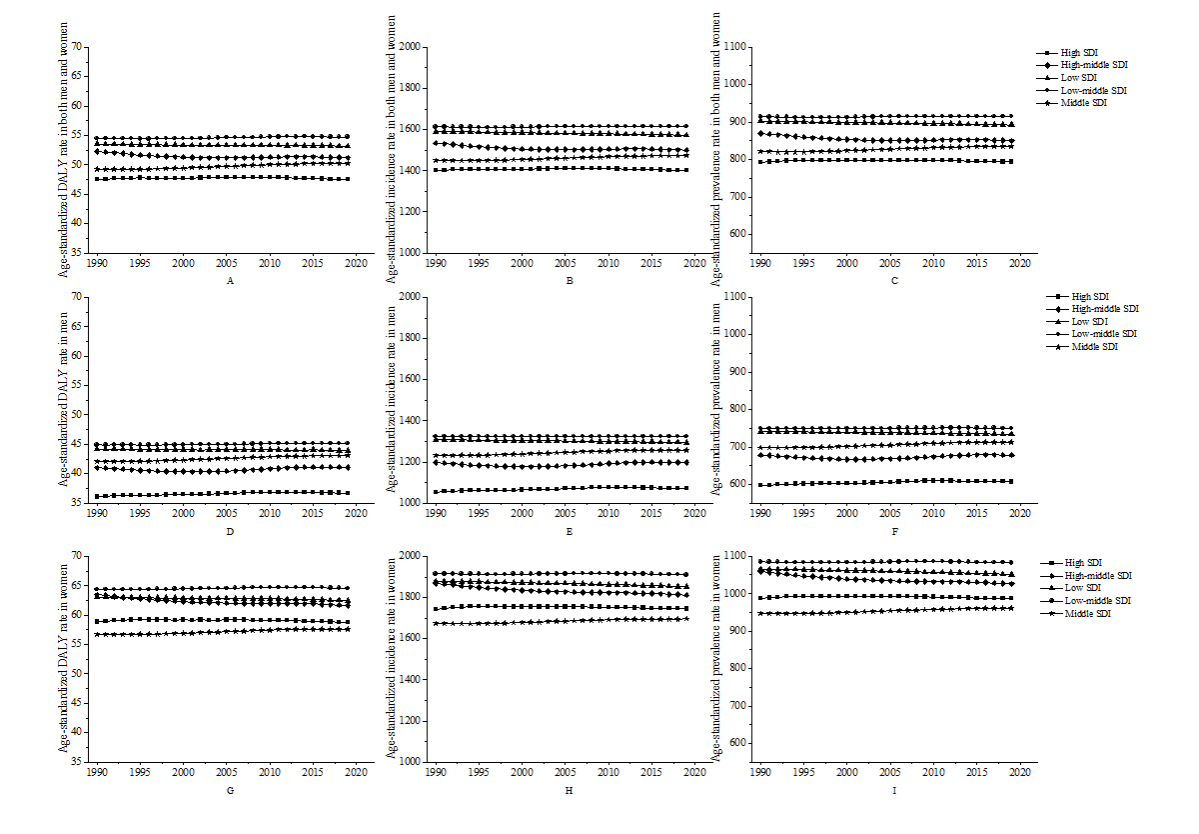
Urticaria age-standardized DALYs, incidence rate, and prevalence rate for both men and women (A, B, C), women (D, E, F), and men (G, H, I) in different SDI areas from 1990 to 2019. DALY: disability-adjusted life year; SDI: Socio-demographic Index.

In 2019, low-middle SDI regions had the highest age-standardized prevalence, incidence, and DALY rates, while high SDI regions had the lowest. Middle SDI regions experienced the most significant percentage changes from 1990 to 2019 in all 3 health indicators. All levels of SDI showed a decline in age-standardized DALY rates over the past 30 years, especially for the high-middle SDI group, which decreased from 52.25 per 100,000 population in 1990 to 51.18 per 100,000 population in 2019. However, the decline in the low-middle SDI group was smaller, from 54.44 per 100,000 population in 1990 to 54.72 per 100,000 population in 2019. Overall, the age-standardized DALY rates for women were higher than those for men in all SDI groups. In terms of different SDI groups, the high SDI group consistently had the lowest age-standardized DALY rates, while the low SDI group consistently had the highest rates. The age-standardized DALY rates for women were consistently higher than those for men, especially in the high SDI and high-middle SDI groups (Figure 2; Multimedia Appendix 1, Table S1).

**Figure 3:**
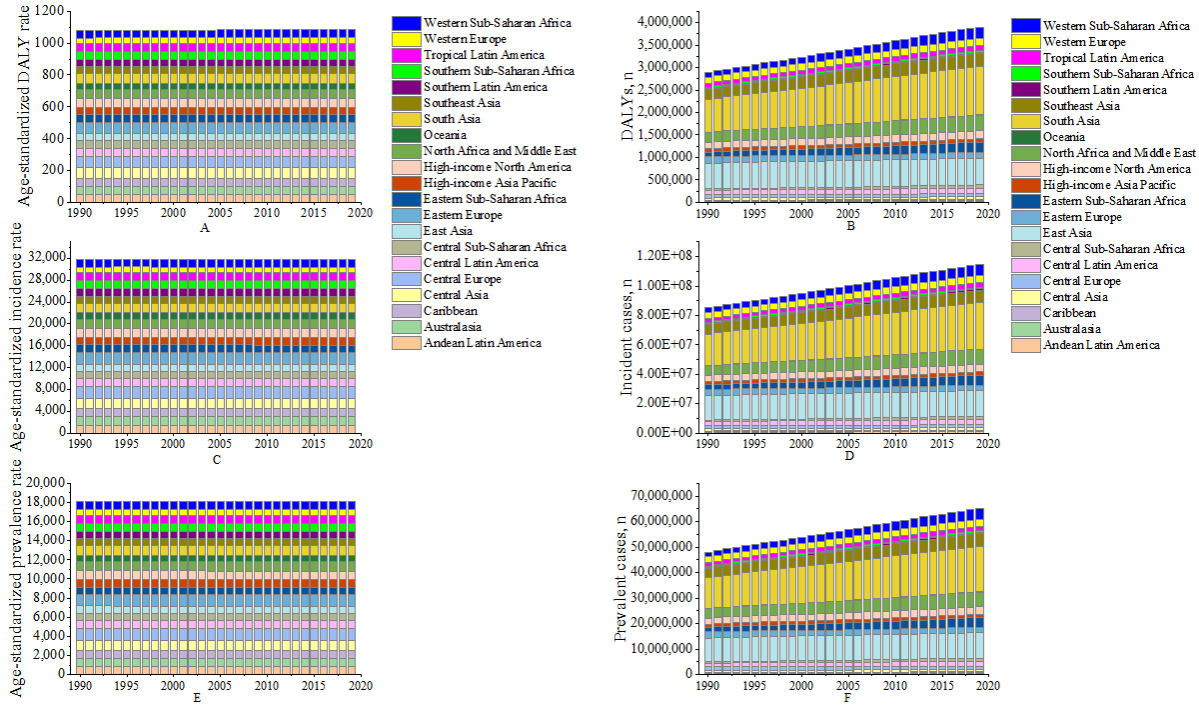
Age-standardized DALY rate (A), number of DALYs (B), incidence rate (C), incident cases (D), prevalence rate (E), and prevalent cases (F) for urticaria in 21 disease burden regions from 1990 to 2019. DALY: disability-adjusted life year.

Incidence rates declined for all SDI groups, with the greatest decrease observed in the high-middle SDI group, from 1534.30 per 100,000 population in 1990 to 1499.49 per 100,000 population in 2019. However, the change in incidence in the low-middle SDI group was not obvious, from 1613.38 per 100,000 population in 1990 to 1613.91 per 100,000 population in 2019. Women had higher incidence rates than men, especially in high SDI and high-middle SDI groups. Prevalence rates remained stable across different SDI levels, with women having higher rates than men. In 2019, Central Europe had the highest age-standardized prevalence, incidence, and DALY rates, while Western Europe had the lowest. From 1990 to 2019, all 21 regions showed stable age-standardized rates but increasing DALY, prevalence, and incidence numbers, with South Asia and East Asia having significantly higher numbers (Figure 2-3; Multimedia Appendix 1, Table S1).

### National Level

**Figure 4:**
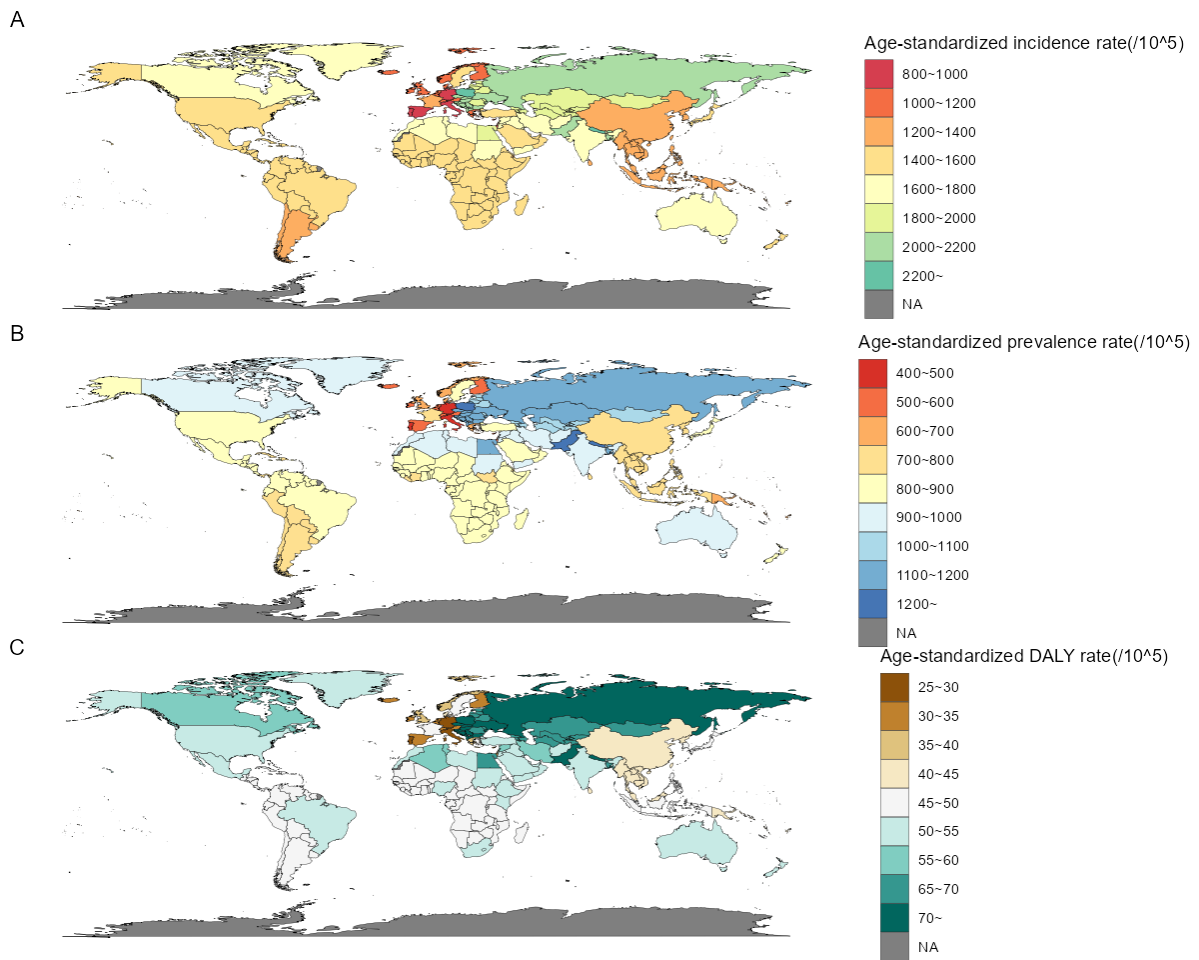
Age-standardized incidence rate (A), age-standardized prevalence rate (B), and age-standardized DALY rate (C) for urticaria per 100,000 population in 2019 in 204 countries and territories. DALY: disability-adjusted life year; NA: not available.

**Figure 5:**
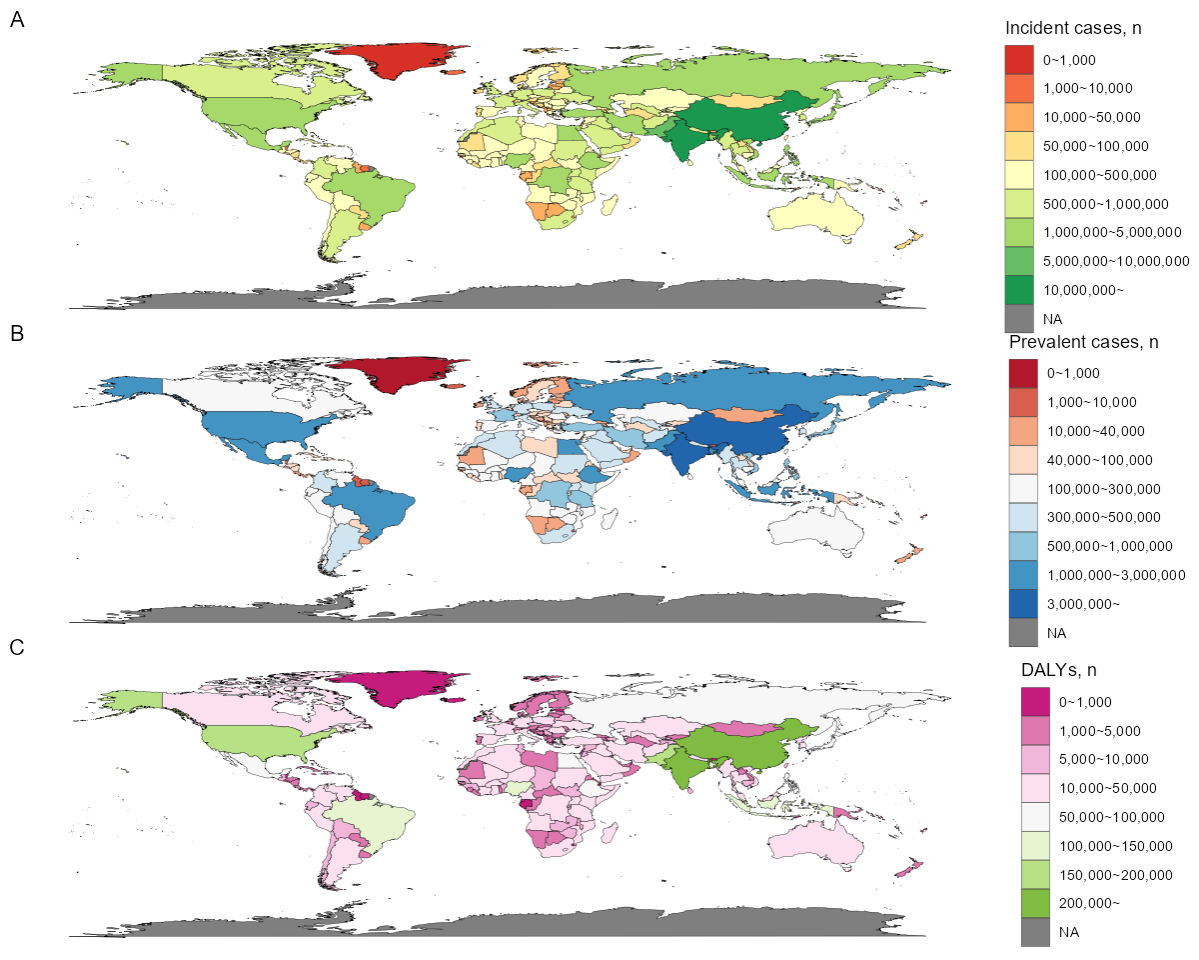
Incident cases (A), prevalent cases (B), and DALY numbers (C) for urticaria in 2019 in 204 countries and territories. DALY: disability-adjusted life year.

The DALYs of urticaria varied significantly between countries and territories. In 2019, the lowest rate, 27.1 (95% uncertainty interval [UI] 17.7-38.1) per 100,000 population, was reported for Portugal, while the highest rate, 92.0 (95% UI 60.3-130.2) per 100,000 population, was reported for Nepal. The data include the DALY numbers for each country and territory in 2019, with India having the highest number at 749,495.9 (95% UI 490,301.3-1,074,876.0), followed by China at 576,544.5 (95% UI 379,886.1-818,597), Pakistan at 180,580.9 (95% UI 117,789.7-263,500.0), and the United States at 153,479.1 (95% UI 102,752.9-213,697.0), among others. The countries and territories with the lowest DALY numbers included Tokelau (0.60, 95% UI 0.39-0.87), Niue (0.67, 95% UI 0.43-0.96), and Nauru (4.74, 95% UI 3.09-6.93). From 1990 to 2019, the annual percentage change in age-standardized DALY rate differed, with the highest decrease in Qatar at –0.0719% (EAPC –0.0719, 95%CI –0.0906 to –0.0532) (Figure 4-5; Multimedia Appendix 1, Table S3).

For incidence, the age-standardized rate and number of new cases also varied widely across countries and territories. In 2019, out of 204 countries and territories, Portugal reported the lowest rate, 818.2 (95% UI 728.6-917.3) per 100,000 population, while Nepal had the highest rate, 2665.5 (95% UI 2904.2-2445.1) per 100,000 population. Incident cases in the 204 countries and territories varied greatly, ranging from as low as 17.8 (95% UI 15.6-20.5) in Tokelau to as high as 22,065,409 (95% UI 19,265,922-25,287,089) in India. Between 1990 and 2019, the annual percentage change in age-standardized incidence rate varied across countries and territories, with Qatar having the highest decrease at –0.0774% (EAPC –0.0774, 95% CI –0.0961 to –0.0587) (Figure 4-5; Multimedia Appendix 1, Table S3).

In terms of prevalence, there was considerable variation in age-standardized rates and the number of cases across countries and territories. Portugal reported the lowest age-standardized prevalence rate, 456.4 (95% UI 406.2-512.2) per 100,000 people in 2019, while Nepal reported the highest rate, 1534.6 (95% UI 1416.2-1662.5) per 100,000 people. Prevalence numbers for the 204 countries and territories ranged widely, from as low as 10.0 (95% UI 8.7-11.5) in Tokelau to as high as 12,552,793 (95% UI 10,916,085-14,379,341) in India. From 1990 to 2019, annual percentage changes in age-standardized prevalence rate varied across countries and territories. Qatar had the highest decrease, with a decline of 0.0795% (EAPC –0.0795, 95% CI –0.0988 to –0.0603) (Figure 4-5; Multimedia Appendix 1, Table S3).

### Age

We analyzed global data on rates and numbers for DALYs, prevalence, and incidence across age groups between 1990 and 2019. Rates were higher in younger age groups, decreased with age, and slightly increased in the oldest groups. Women had higher rates than men. A general increase in rates and numbers was observed across age groups between 1990 and 2019 that was more pronounced in younger populations and women. The largest increases occurred in age groups younger than 40 years, while the smallest increases were in groups older than 70 years. In both age groups, women consistently had higher numbers for DALYs, prevalence, and incidence, indicating an overall rising trend in disease burden, prevalence, and incidence (Multimedia Appendix 2, Figure S1).

Regional and SDI-level urticaria disease burden were also analyzed, focusing on rates and numbers for DALYs, prevalence, and incidence across age groups and genders. The youngest age group generally had the highest rates and numbers, decreasing with age. Women consistently had higher rates and numbers across most age groups and regions. Significant regional and SDI-level variations existed, with regions like South Asia, Western sub-Saharan Africa, North Africa, and the Middle East having higher burdens. Central and Eastern Europe had higher rates and numbers than Western Europe. DALY numbers decreased with age across SDI regions and genders, with women having higher numbers. DALY rate trends were similar, but differences between SDI regions and genders were less pronounced (Multimedia Appendix 2, Figures S2-S5).

At the SDI level, urticaria prevalence rates were analyzed across age groups, genders, and SDI regions. The rates were highest in the low-middle SDI group and generally decreased with age, with women consistently having higher rates. High and high-middle SDI groups had lower prevalence rates than low and low-middle groups. The prevalence number also decreased with age and was higher in women. High SDI and high-middle SDI regions had lower prevalence numbers (Multimedia Appendix 2, Figure S6).

Urticaria incidence rates varied across age groups and SDI regions, with women generally having higher rates. The highest incidence rate was in children younger than 5 years, and rates decreased with age. Low-middle SDI regions had the highest incidence rates, while middle SDI regions had the lowest. Incidence numbers followed similar trends, with higher numbers in low SDI and low-middle SDI regions and a decrease with age. The incidence was consistently higher in women across all groups (Multimedia Appendix 2, Figure S7).

## Discussion

Our study examines global, regional, and national urticaria trends by age, gender, and SDI regions. Women had higher prevalence, incidence, and DALYs from 1990 to 2019. Urticaria rates were highest in low-middle SDI regions and lowest in high SDI regions. Central Europe had the highest age-standardized rates, while Western Europe had the lowest. Portugal had the lowest rate in 2019 and Nepal the highest. Annual age-standardized rates fluctuated over 30 years. Women had higher urticaria rates across all age categories. Between 1990 and 2019, disease burden, prevalence, and incidence increased across most age categories, particularly in younger people and women.

One of the main findings in our study is the higher burden of urticaria among women compared to men. This observation is consistent with previous studies that have reported a higher prevalence of autoimmune diseases in women [15,16]. The higher rates of urticaria in women may be attributed to several factors, including hormonal differences, genetic predispositions, and environmental exposures [17-19]. Hormonal fluctuations, particularly estrogen levels, have been implicated in the regulation of immune responses and may contribute to the increased susceptibility of women to autoimmune diseases [20,21]. Genetic factors may also play a role, as certain genes related to immune function are located on the X chromosome, potentially resulting in a higher risk of autoimmune diseases in women [22]. Further research is needed to better understand the underlying mechanisms and develop targeted interventions to reduce the burden of urticaria in affected individuals [23-25].

The observed increase in the global burden of urticaria from 1990 to 2019 is a concerning trend that warrants further investigation. Several factors could potentially contribute to this increase, such as changes in environmental exposures, lifestyle factors, and diagnostic practices. Increased exposure to allergens and irritants, such as air pollution and chemical substances, may have contributed to the rise in urticaria prevalence [1,26]. Additionally, changes in lifestyle factors, such as diet, stress, and sedentary behavior, may also have contributed to the increase in urticaria cases [27,28]. Lastly, improvements in diagnostic practices and increased awareness of the condition may have led to a higher detection rate of urticaria cases over time [29,30].

Our findings also highlight substantial regional variations in the burden of urticaria, with low-middle SDI regions exhibiting the highest rates and high SDI regions displaying the lowest. These disparities can be attributed to differences in socioeconomic conditions, health care infrastructure, and access to care, which may influence the overall disease burden in these regions [31-34]. Moreover, regional variations in environmental exposures, such as air pollution and allergen prevalence, could contribute to the observed differences in urticaria burden across regions [35-38].

At the national level, we found significant variation in urticaria’s disease burden, with age-standardized rates and numbers of DALYs, incidence, and prevalence differing substantially across countries and territories. These findings highlight the importance of understanding the unique factors that contribute to the burden of urticaria in different countries and territories and the need for tailored interventions to address these factors. For instance, countries and territories with a high burden of urticaria may benefit from increased investment in health care infrastructure, improved access to care, and targeted public health interventions to reduce exposure to environmental risk factors [39-44].

Our study also revealed that the burden of urticaria is generally higher in younger age groups and decreases with age, with a slight increase observed in the oldest age groups. This pattern may be partially explained by differences in immune system function and regulation across different age groups, with younger individuals potentially being more susceptible to immune-mediated conditions such as urticaria [45,46]. Additionally, the increased burden of urticaria in the youngest age groups may be related to the high frequency of infections in children, which can trigger urticaria in susceptible individuals [47,48]. The slight increase in urticaria burden among the oldest age groups may be attributed to age-related changes in the immune system, known as immunosenescence, which can result in a higher risk of autoimmune diseases [1,49].

Our study has several strengths, including the use of a comprehensive data set spanning 3 decades and covering multiple aspects of urticaria burden at the global, regional, and national levels. However, several limitations should also be acknowledged. First, the quality and availability of data on urticaria burden may vary across countries and territories and time periods, potentially affecting the accuracy of our estimates. Second, our study primarily focused on a descriptive analysis of trends in urticaria burden and did not explore the underlying causal factors contributing to these trends. Future research should aim to identify the specific risk factors and mechanisms that contribute to the observed disparities in urticaria burden across different populations and age groups.

In conclusion, our findings highlight the increasing burden of urticaria at the global, regional, and national levels, with substantial disparities observed across age, gender, and SDI groups. The higher burden of urticaria among women, the increasing burden among younger populations, and the regional variations in disease burden underscore the need for targeted interventions and policies to address this growing public health concern. Further research is required to identify the specific risk factors and mechanisms underlying the observed disparities in urticaria burden and inform the development of effective interventions and strategies to reduce the global burden of this condition.

## Appendix

Multimedia Appendix 1 Supplementary Tables S1-S3.

Multimedia Appendix 2 Supplementary Figures S1-S7.

## Abbreviations

DALY: disability-adjusted life year
EAPC: age-standardized estimated annual percentage change
GBD: Global Burden of Disease
IHME: Institute for Health Metrics and Evaluation
SDI: Socio-demographic Index
UI: uncertainty interval

## Data Availability

The data sets generated during and/or analyzed during this study are available in the Global Burden of Disease (GBD) study’s public database [50].
