# Supplementary material for: Burden of and Trends in Urticaria Globally, Regionally, and Nationally from 1990 to 2019: Systematic Analysis
Source: JMIR Public Health Surveill. 2023 Oct 26;9:e50114. doi: 10.2196/50114 (PMC10636626; doi:10.2196/50114)
Supplement: Multimedia Appendix 1 [file publichealth_v9i1e50114_app1.docx]

**Table S1 Prevalence cases, incidence cases, and disability-adjusted life years (DALYs) for urticaria in 2019 and percentage change in age standardized rates (ASRs) per 100,000, by SID and global burden of disease region, from 1990 to 2019.**

|  | **Prevalence** | | |  | **Incidence** | | |  | **DALYs** | | |
| --- | --- | --- | --- | --- | --- | --- | --- | --- | --- | --- | --- |
| **location** | **Number**  **（95% UI）** | **Age-standardized**  **Rate**  **（95% UI）** | **Percentage**  **Change in ASRs from 1990 to 2019 （95% CI）** |  | **Number**  **（95% UI）** | **Age-standardized**  **Rate**  **（95% UI）** | **Percentage**  **Change in ASRs from 1990 to 2019 （95% CI）** |  | **Number**  **（95% UI）** | **age-standardized**  **Rate**  **（95% UI）** | **Percentage**  **Change in ASRs from 1990 to 2019 （95% CI）** |
| High-middle SDI | 11005530 (9740649-12294294.8) | 849.6 (744.5-960.9) | -0.059 (-0.0756-0.0423) |  | 19322325.1 (17180176-21506715.1) | 1499.5 (1317.8-1696.8) | -0.0534 (-0.0693-0.0376) |  | 656089.3 (432160.1-936970.6) | 51.2 (33.5-73.7) | -0.047 (-0.0638-0.0301) |
| High SDI | 7343934.1 (6742022.2-7967165.8) | 793.4 (722.9-868.7) | -0.0014 (-0.0098-0.007) |  | 12895273.6 (11796168-14024348.7) | 1402 (1275.9-1540) | -4e-04 (-0.0091-0.0083) |  | 433027.6 (285343.5-609777.2) | 47.5 (31.3-67.6) | -4e-04 (-0.0103-0.0095) |
| Low-middle SDI | 16317695.3 (14178150.7-18706227.2) | 914.5 (798.3-1045.9) | 0.0099 (0.0071-0.0128) |  | 28743355.4 (25132467.9-32898741.7) | 1613.9 (1413.8-1838.8) | 0.0088 (0.0059-0.0116) |  | 979311.1 (639278.3-1420447.6) | 54.7 (35.8-79.1) | 0.0281 (0.0243-0.032) |
| Low SDI | 11276098.2 (9630528.5-13227738.4) | 892.1 (781-1018.5) | -0.0372 (-0.0383-0.0362) |  | 19996940.7 (17122620.4-23307907.6) | 1572.5 (1384.6-1782.1) | -0.0373 (-0.0382-0.0364) |  | 678840.9 (443143.6-992715.1) | 53.2 (34.9-76.6) | -0.0173 (-0.0196-0.0149) |
| Middle SDI | 19161780.6 (16869909-21553729.4) | 834.6 (731.3-946.7) | 0.0741 (0.0691-0.0791) |  | 33689536.8 (29634184.6-38028973.6) | 1473 (1294.6-1671.1) | 0.0725 (0.0675-0.0775) |  | 1149480.9 (752418-1643055.5) | 50.2 (32.8-72.2) | 0.0836 (0.078-0.0892) |
| Global | 65139886.6 (57516618-73498729.7) | 865.5 (761.8-980.6) | 0.0234 (0.0192-0.0276) |  | 114708912.2 (101309687.1-129286333.7) | 1527.5 (1346.2-1726.5) | 0.022 (0.0176-0.0263) |  | 3898838.6 (2554225.4-5584365.6) | 51.9 (34-75.1) | 0.0338 (0.0287-0.0388) |
| Andean Latin America | 509792.4 (443612.4-584260.7) | 799.6 (698.1-916.3) | -0.0069 (-0.0074-0.0063) |  | 899584.5 (788300.7-1024420.2) | 1411.1 (1239.6-1602.6) | -0.0066 (-0.0072-0.0061) |  | 30723.5 (19985.1-44502.2) | 48.1 (31.4-69.8) | 0.0078 (0.0055-0.0101) |
| Australasia | 248806.1 (221224-279645.3) | 912.7 (800.5-1034.4) | 0.0069 (0.0052-0.0086) |  | 434983.4 (387502.6-489452.5) | 1604.4 (1411.7-1823.9) | 0.0065 (0.0048-0.0082) |  | 14694.8 (9608.9-21046.6) | 54.5 (35.3-78.2) | 0.011 (0.0076-0.0144) |
| Caribbean | 364983 (320457.3-414147.4) | 800.8 (699.1-917.7) | -0.0035 (-0.004-0.0031) |  | 642654.2 (568334.3-725955.9) | 1413.1 (1241.4-1605) | -0.0034 (-0.0039-0.003) |  | 21826.7 (14363.9-31358.7) | 48 (31.5-69.1) | -0.0069 (-0.0084-0.0054) |
| Central Asia | 1032734.5 (892207.9-1188557.6) | 1097.3 (949.1-1255.3) | -0.0113 (-0.012-0.0107) |  | 1816768 (1569818.2-2089037.6) | 1932.4 (1678.2-2216.4) | -0.0113 (-0.012-0.0106) |  | 62416.7 (40782.9-90250) | 66.2 (43.3-96) | -0.005 (-0.0063-0.0036) |
| Central Europe | 1131039.7 (1024499.8-1249111.8) | 1189.3 (1054.2-1337.9) | -0.013 (-0.0187-0.0074) |  | 1982799.6 (1795870-2190677) | 2097.6 (1862.6-2363.4) | -0.0122 (-0.0177-0.0068) |  | 67169.3 (44596.1-95460.2) | 71.9 (47.2-103.4) | -0.0034 (-0.0084-0.0017) |
| Central Latin America | 2018903 (1767759.5-2298139.8) | 825.1 (722.9-944.6) | -0.0044 (-0.0048-0.004) |  | 3553871.4 (3114457.8-4036689.6) | 1456.2 (1277.5-1656.1) | -0.0044 (-0.0048-0.004) |  | 121438.7 (79078.5-175939.7) | 49.7 (32.4-72.3) | 0 (-0.0013-0.0013) |
| Central Sub-Saharan Africa | 1195622.8 (1017182.1-1408618.6) | 802 (700.2-918.8) | -8e-04 (-0.0015-1e-04) |  | 2121637.6 (1809186.9-2497240.5) | 1415.3 (1243.5-1607.4) | -8e-04 (-0.0014-1e-04) |  | 71958.9 (46757.3-105267.1) | 47.8 (31.5-68.9) | 0.0219 (0.0178-0.0261) |
| East Asia | 9959598 (8771338.1-11131861.4) | 737.5 (644.6-838.5) | -0.0105 (-0.0113-0.0097) |  | 17498730.8 (15478051.1-19510976.9) | 1303.6 (1143.2-1488.5) | -0.0105 (-0.0114-0.0096) |  | 595886.5 (392144.5-845623.1) | 44.6 (29.1-64.8) | 0.0022 (6e-04-0.0039) |
| Eastern Europe | 2113927 (1875050.2-2377245.1) | 1160.3 (1005.3-1337) | -0.0035 (-0.0047-0.0023) |  | 3703011.3 (3287930.1-4159238.6) | 2043.6 (1771.5-2355.2) | -0.0032 (-0.0044-0.002) |  | 125843.9 (82383.2-180930.6) | 70.1 (46-102.1) | 0.0068 (0.005-0.0085) |
| Eastern Sub-Saharan Africa | 3808731.9 (3245202.9-4490808.2) | 819.4 (717.7-938.1) | -0.0028 (-0.0034-0.0023) |  | 6763691.6 (5782086.5-7943167.2) | 1445.9 (1270.7-1644.7) | -0.003 (-0.0035-0.0024) |  | 230139.9 (150650.3-338951) | 49 (32.1-71.1) | 0.0238 (0.0208-0.0269) |
| High-income Asia Pacific | 1259639.1 (1120235.8-1402664.9) | 785.5 (688.1-896.9) | -0.0249 (-0.0264-0.0235) |  | 2203654.1 (1973191.7-2445546) | 1386.1 (1221.1-1582.1) | -0.0242 (-0.0258-0.0227) |  | 74570 (49254.8-105635.9) | 47.4 (31.1-68.4) | -0.0149 (-0.0172-0.0126) |
| High-income North America | 2906353.5 (2748972.1-3069013.1) | 895.5 (841.3-955) | 0.0075 (-0.0038-0.0187) |  | 5119005.5 (4812424.7-5442408.7) | 1585.9 (1477.3-1705) | 0.0108 (-0.0018-0.0233) |  | 171109 (114326.9-239859.8) | 53.5 (35.5-75.6) | 0.0018 (-0.0116-0.0153) |
| North Africa and Middle East | 5832733.1 (5071054.1-6635879.9) | 950.5 (828.9-1075.5) | 0.0042 (0.0022-0.0062) |  | 10257547.3 (8982582-11722172) | 1673.5 (1467.4-1901.2) | 0.0037 (0.0018-0.0056) |  | 350946.2 (229607.5-508231.2) | 57 (37.4-82.8) | 0.0094 (0.0077-0.0111) |
| Oceania | 97255.1 (84284-112227.5) | 694 (609.9-791) | -0.0012 (-0.0016-8e-04) |  | 172994.7 (150031.7-201075) | 1226.7 (1079.6-1402.7) | -0.0013 (-0.0017-9e-04) |  | 5853.3 (3843.8-8629.9) | 41.4 (27.1-59.8) | 2e-04 (-0.0018-0.0023) |
| South Asia | 17852322.3 (15540849-20450848.8) | 989.5 (863.2-1133.9) | 0.0309 (0.0291-0.0328) |  | 31399687.3 (27475463.4-35943203) | 1746.4 (1533.8-1994) | 0.0307 (0.0289-0.0325) |  | 1068940.9 (696805.2-1546075.7) | 59.1 (38.6-85.3) | 0.0518 (0.0497-0.054) |
| Southeast Asia | 4943703.8 (4321517.2-5612246.3) | 741.7 (648.8-843) | 0.0074 (0.0071-0.0078) |  | 8715606.9 (7636537.8-9879226.2) | 1312.3 (1151.9-1492.8) | 0.0077 (0.0073-0.008) |  | 297242.9 (196270.5-423283.2) | 44.6 (29.4-63.7) | 0.0254 (0.0236-0.0271) |
| Southern Latin America | 483941.6 (426009.7-546648) | 762.7 (667.1-867.6) | -0.0045 (-0.005-0.004) |  | 850017.1 (752461.7-955430.6) | 1345.3 (1185.6-1530.6) | -0.0046 (-0.0051-0.0041) |  | 28928.2 (18987.6-41298.2) | 45.8 (30-66.2) | 1e-04 (-0.0024-0.0026) |
| Southern Sub-Saharan Africa | 667269 (580500.1-766832.9) | 835.8 (731.3-954.9) | -0.0072 (-0.0106-0.0037) |  | 1176908.5 (1029405.4-1345264.2) | 1475 (1292.4-1675.9) | -0.007 (-0.0104-0.0036) |  | 40035 (25993.7-58297.4) | 50 (32.6-72.5) | -0.0101 (-0.0115-0.0088) |
| Tropical Latin America | 1789675.5 (1574377.3-2023147.2) | 846.7 (741-968.2) | -0.0024 (-0.0027-0.0021) |  | 3145257.7 (2769115.5-3558308.9) | 1494.3 (1305.4-1704.9) | -0.0024 (-0.0027-0.0021) |  | 106852.1 (69841.2-154007.6) | 50.8 (33.1-73.5) | 0.0073 (0.0052-0.0093) |
| Western Europe | 2653796.3 (2384320.3-2924430.4) | 588 (522.9-653.8) | 0.0112 (0.0091-0.0132) |  | 4660503.9 (4201191.5-5158333.1) | 1040.2 (927.3-1155.9) | 0.0115 (0.0094-0.0137) |  | 155143.4 (103509.7-217649.1) | 34.9 (23-49.4) | 0.0161 (0.0135-0.0187) |
| Western Sub-Saharan Africa | 4269059.1 (3638323.4-5037789.4) | 825.3 (723-944.5) | 0.0137 (0.0099-0.0175) |  | 7589996.7 (6492713-8916267.7) | 1456.3 (1277.9-1656.2) | 0.0137 (0.0101-0.0174) |  | 257118.5 (168026.7-376269.7) | 49.3 (32.3-71.5) | 0.031 (0.0247-0.0373) |

**Table S2 Prevalence cases, incidence cases, and disability-adjusted life years (DALYs) for urticaria in 1990 per 100,000, by SID and global burden of disease region, from 1990 to 2019.**

|  | **Prevalence** | |  | **Incidence** | |  | **DALYs** | |
| --- | --- | --- | --- | --- | --- | --- | --- | --- |
| **location** | **Number**  **（95% UI）** | **age-standardized**  **Rate**  **（95% UI）** |  | **Number**  **（95% UI）** | **age-standardized**  **Rate**  **（95% UI）** |  | **Number**  **（95% UI）** | **age-standardized**  **Rate**  **（95% UI）** |
| High-middle SDI | 9889966.3  (8687818.9-11195304.2) | 869.9  (762.3-985.8) |  | 17406649.5 (15302501.2-19715926.8) | 1534.3  (1346.7-1735.5) |  | 594152.8 (387212.6-856327.3) | 52.2  (34.1-75.2) |
| High SDI | 6225585.4  (5662982.6-6804671.1) | 792.6  (719-872) |  | 10955581 (9951294.6-12005899.8) | 1399.7  (1269.5-1542.4) |  | 370391.8 (244053.2-522773.6) | 47.5  (31.2-67.4) |
| Low-middle SDI | 11518842.9  (9820743.6-13495995.1) | 914.0  (797.8-1044.4) |  | 20474533.3 (17521046.9-23811174.9) | 1613.4  (1413.4-1838.7) |  | 692177.7 (450787.7-1008459.3) | 54.4  (35.6-78.5) |
| Low SDI | 5569453  (4743831.3-6541214.3) | 902.2  (791.9-1028.3) |  | 9965039.4 (8495452.7-11692241.5) | 1590.4  (1401.3-1801.3) |  | 334010.1 (218227.5-489455.3) | 53.5  (35.1-77.4) |
| Middle SDI | 14861379.7 (12846345.4-17123578.5) | 820.6  (718.2-930.2) |  | 26280207.3 (22858796.8-30326536.3) | 1448.9  (1273-1645.7) |  | 897567 (584515.9-1299659.9) | 49.2  (32.1-70.7) |
| Global | 48088422.3 (41843377.8-54882802.3) | 862.6  (759.5-975.8) |  | 85123147.9 (74624896.1-96974475.9) | 1522.7  (1343-1721.8) |  | 2889695.3 (1881485.9-4200997.5) | 51.6  (33.8-74.2) |
| Andean Latin America | 335748.1  (287187-392206.9) | 800.8  (699.1-917.8) |  | 595871.3  (511486.2-695478.9) | 1413.1  (1241.4-1605.5) |  | 20290.1  (13291.1-29655.9) | 48.0  (31.5-69.2) |
| Australasia | 179573.5  (158414.6-203284.3) | 910.3  (798.1-1030.6) |  | 314711.6  (278596.6-355479.6) | 1600.3  (1407.9-1818.3) |  | 10667.4  (6964.2-15115.8) | 54.3  (35.7-77.5) |
| Caribbean | 293835.9  (253707.5-339629.7) | 801.4  (699.6-918.5) |  | 520222.9  (452628-598188.5) | 1414.2  (1242.4-1606.9) |  | 17706.5  (11547.5-25817.9) | 48.1  (31.5-69.8) |
| Central Asia | 845555.7  (718669.2-988408.1) | 1101.0 (952.5-1259.8) |  | 1495185.7 (1279437.9-1747458.7) | 1938.9  (1683.7-2223.4) |  | 51231.8  (33493.6-74614.6) | 66.3  (43.6-95.8) |
| Central Europe | 1363546.1  (1210142.2-1534333) | 1198.2 (1049.5-1364.1) |  | 2387886.4 (2120092.9-2685755.7) | 2112.4  (1849.1-2403.1) |  | 81794.8 (53933.2-118171.5) | 72.3  (47.5-104.2) |
| Central Latin America | 1483735.6  (1272961.5-1734328.8) | 826.0  (723.6-945.8) |  | 2630184.9 (2260517.3-3069643.4) | 1457.7  (1278.3-1658.1) |  | 89957.4 (58494.7-131864.7) | 49.6  (32.3-72) |
| Central Sub-Saharan Africa | 523483.5  (443615.8-620372) | 802.2  (700.3-919.3) |  | 940493.6  (795344.6-1114474.2) | 1415.5  (1243.8-1607.7) |  | 31361.3  (20644.9-46005.1) | 47.5  (31.4-68.7) |
| East Asia | 9283862.4  (8037493.4-10639098) | 740.1  (646.6-841.8) |  | 16384722.2 (14240973.8-18876763.9) | 1308.2  (1147.4-1493.8) |  | 561250  (364288-810674.3) | 44.6  (29.2-64.7) |
| Eastern Europe | 2449286.3  (2148497.7-2778046.6) | 1162.6 (1007.4-1340.1) |  | 4283872.5 (3757382.5-4867607.9) | 2047.5  (1775.2-2358.2) |  | 146607.4 (95797.1-211523.6) | 70.0  (45.7-102.1) |
| Eastern Sub-Saharan Africa | 1834297.8  (1553194.1-2175588.4) | 819.7  (717.8-938.4) |  | 3289101.3 (2797498.8-3886828.7) | 1446.6  (1271-1646.2) |  | 110306.9 (71728.9-162984.1) | 48.8  (31.8-70.8) |
| High-income Asia Pacific | 1282505.7  (1131228.2-1444597.6) | 791.8  (694.2-902.9) |  | 2246408.5 (1986836.3-2540252.9) | 1397.0  (1229.6-1593.9) |  | 76764.3 (50531.2-110521.8) | 47.7  (31.3-69) |
| High-income North America | 2348611.9  (2204493.9-2496370.2) | 885.4  (829.4-946.3) |  | 4146143.7 (3876453.3-4426138.4) | 1565.1  (1455.3-1687.5) |  | 139663.5 (92687.9-197132.9) | 53.0  (35.1-74.9) |
| North Africa and Middle East | 3653590  (3118221.2-4235389.5) | 950.0  (829-1075.6) |  | 6480041.8 (5553999.4-7508090.2) | 1673.1  (1467.4-1903.5) |  | 220985.6 (144152.2-323560.9) | 56.9 (37.1-82) |
| Oceania | 48645.3  (41960.6-56588.7) | 694.0  (609.9-791.1) |  | 86663.3  (74412.2-101154.7) | 1226.8  (1079.6-1402.9) |  | 2934.5  (1910.8-4278) | 41.5  (27.2-59.3) |
| South Asia | 12114820.9 (10391954.7-14214012.8) | 981.1  (856.2-1122.7) |  | 21509608.3 (18466973.3-25045546.8) | 1731.7  (1521.6-1977.9) |  | 726277 (473731.7-1055685.6) | 58.3  (38.3-83.8) |
| Southeast Asia | 3648495.8  (3144814.6-4208078.4) | 740.2  (647.7-841.3) |  | 6474985.2 (5604466.7-7524301.7) | 1309.4  (1148.8-1490) |  | 220182.8 (145247.1-315169.9) | 44.3  (29.3-63.4) |
| Southern Latin America | 383072.6  (334036.3-437149.5) | 763.7  (668.1-868.7) |  | 675421.9  (592634.5-769259.9) | 1347.3  (1187.2-1532.9) |  | 23038.1  (15073.3-33464) | 45.9  (29.9-66.5) |
| Southern Sub-Saharan Africa | 479122  (410532-559111.8) | 837.4  (732.8-957.3) |  | 848270  (730075-988632.7) | 1477.7  (1294.8-1679.8) |  | 28938.1  (18881.3-42307.8) | 50.1  (32.8-72.7) |
| Tropical Latin America | 1367565.6  (1179243.9-1586418.4) | 847.2  (741.4-969) |  | 2409965.1 (2080935.9-2784198.5) | 1495.2  (1305.8-1706.1) |  | 82324.2 (53547.9-119971.2) | 50.6  (33.2-73.3) |
| Western Europe | 2325241  (2079017.7-2565848.7) | 586.2  (521.4-652.1) |  | 4094972.3 (3669431.1-4533175.7) | 1036.9  (924.9-1152.7) |  | 136808.9  (90836.4-191363) | 34.8  (22.9-49.1) |
| Western Sub-Saharan Africa | 1843826.6  (1564226.3-2181101.7) | 822.5  (720.5-941.4) |  | 3308415.2 (2816240.4-3912632.8) | 1451.4  (1273.6-1651.1) |  | 110604.6 (72061.7-162297.9) | 48.9  (32-70.8) |

**Table S3 Prevalence cases, incidence cases, and disability-adjusted life years (DALYs) for urticaria in 2019 and percentage change in age standardised rates (ASRs) per 100000, in 204 countries and territories, from 1990 to 2019.**

|  | **Prevalence** | | |  | **Incidence** | | |  | **DALYs** | | |
| --- | --- | --- | --- | --- | --- | --- | --- | --- | --- | --- | --- |
| **location** | **Number**  **（95% UI）** | **age-standardized**  **Rate**  **（95% UI）** | **precentage**  **Change in ASRs from 1990 to 2019 （95% CI）** |  | **Number**  **（95% UI）** | **age-standardized**  **Rate**  **（95% UI）** | **precentage**  **Change in ASRs from 1990 to 2019**  **（95% CI）** |  | **Number**  **（95% UI）** | **age-standardized**  **Rate**  **（95% UI）** | **precentage**  **Change in ASRs from 1990 to 2019**  **（95% CI）** |
| Afghanistan | 401580.4 (339619.7-473101.2) | 915.3 (800-1044.6) | -0.0154 (-0.019--0.0118) |  | 715405.8 (605088.2-844090.7) | 1614.6 (1416.5-1844.5) | -0.0151 (-0.0186--0.0116) |  | 24206.6 (15639-35698.1) | 54.3 (35.3-78.5) | 0.0048 (3e-04-0.0093) |
| Albania | 27411.9 (24285.5-30966.5) | 1165.1 (1012.8-1344.1) | 0.0149 (0.0068-0.0231) |  | 48222.7 (42760-54420.1) | 2052.7 (1779-2371.2) | 0.015 (0.0069-0.0231) |  | 1639.2 (1080.3-2367.7) | 70.4 (46.5-102.9) | 0.0224 (0.0132-0.0316) |
| Algeria | 386122.7 (335833.7-442913.5) | 915.7 (799.9-1045.9) | -0.0053 (-0.0056--0.0049) |  | 681696.3 (598197.3-776383.7) | 1615.3 (1417-1844.3) | -0.0053 (-0.0056--0.0049) |  | 23256.8 (15163.9-33214) | 55.1 (36.2-79.3) | -0.0021 (-0.0048-5e-04) |
| American Samoa | 390.9 (340.7-449.6) | 695.5 (611.4-792.8) | 0.0042 (0.0039-0.0046) |  | 689.8 (602.9-797.3) | 1229.4 (1081.9-1405.5) | 0.0042 (0.0038-0.0045) |  | 23.5 (15.3-34.2) | 41.6 (27.1-60.2) | -0.0014 (-0.0049-0.002) |
| Andorra | 499.8 (441.1-559.6) | 572 (503.9-642.6) | 0.0574 (0.0472-0.0675) |  | 876.5 (774.9-985.1) | 1012.3 (896.1-1132.6) | 0.0565 (0.0463-0.0667) |  | 29.3 (19.2-41.5) | 34 (22.1-48.8) | 0.0589 (0.0487-0.0692) |
| Angola | 281387.6 (239109-332813.2) | 804.7 (702.5-922) | 0.0233 (0.0213-0.0253) |  | 499876.1 (425082.4-591526.1) | 1419.8 (1247.6-1613.3) | 0.0232 (0.0212-0.0251) |  | 17021.2 (11111.6-24903.8) | 48.1 (31.8-69.4) | 0.0377 (0.0337-0.0416) |
| Antigua and Barbuda | 648.6 (572.8-730.4) | 801.3 (699.6-918.3) | -0.0143 (-0.0197--0.0088) |  | 1137.2 (1003.8-1278.9) | 1414 (1242.4-1606.5) | -0.0139 (-0.0193--0.0085) |  | 38.7 (25.8-55.7) | 48.2 (31.9-70.2) | -0.0122 (-0.0182--0.0062) |
| Argentina | 330466.4 (291015.7-373980.4) | 763.1 (667.6-868.1) | -0.0027 (-0.0036--0.0018) |  | 580725.4 (512574.9-655152.7) | 1346.1 (1186.3-1531.7) | -0.0028 (-0.0037--0.002) |  | 19771 (12955.9-28398.7) | 45.8 (30.1-66.1) | 0.0023 (-0.0011-0.0057) |
| Armenia | 29742.4 (26269.5-33656.9) | 1098.6 (950.7-1257.4) | -0.0059 (-0.0101--0.0017) |  | 52098.2 (45945.4-58594.8) | 1934.7 (1680.6-2220.1) | -0.0062 (-0.0105--0.002) |  | 1780 (1165.5-2556.9) | 66.3 (43.3-96.2) | -0.0018 (-0.0083-0.0047) |
| Australia | 211241 (187217.8-239553.4) | 914.9 (801.2-1044.8) | 0.0036 (0.0022-0.005) |  | 368966.4 (327841.3-416051.6) | 1607 (1412.3-1833.1) | 0.0035 (0.0021-0.0049) |  | 12476.1 (8132.4-17867.9) | 54.7 (35.3-78.2) | 0.0066 (0.0031-0.0101) |
| Austria | 53648.9 (47646.5-59694.1) | 576.6 (507.9-647) | -0.0172 (-0.0203--0.0142) |  | 94288.6 (83945.6-104894) | 1020.2 (903.1-1141.4) | -0.0166 (-0.0198--0.0135) |  | 3138.3 (2079.8-4394.1) | 34.2 (22.5-48.3) | -0.0121 (-0.017--0.0072) |
| Azerbaijan | 103495.9 (90568-118269.4) | 1094.5 (946.7-1252.3) | -0.0271 (-0.0285--0.0256) |  | 181222.5 (158855.3-205334.2) | 1927.4 (1674-2211.4) | -0.0271 (-0.0286--0.0257) |  | 6245.4 (4082.5-9014) | 66.2 (43.2-96) | -0.0234 (-0.0255--0.0212) |
| Bahamas | 2802.1 (2468.3-3157.2) | 803 (700.9-920) | 0.0024 (0.0017-0.0031) |  | 4903.3 (4326.3-5530.9) | 1416.9 (1245-1609.5) | 0.0023 (0.0016-0.003) |  | 167.8 (110.6-242.4) | 48.3 (31.7-69.9) | -1e-04 (-0.0023-0.0022) |
| Bahrain | 11647 (10188.4-13263.7) | 897.2 (783.4-1025.4) | -0.0266 (-0.034--0.0193) |  | 20389.9 (17922.2-23105.9) | 1583.1 (1385.1-1808.9) | -0.0265 (-0.0337--0.0192) |  | 697.5 (452.3-1009.6) | 54 (35-78.3) | -0.0151 (-0.022--0.0082) |
| Bangladesh | 1824231.3 (1584926.3-2083169.4) | 1155.2 (1007.4-1320.1) | 0.0405 (0.0352-0.0457) |  | 3199988.4 (2799923.8-3649823.5) | 2036.2 (1779.6-2317.6) | 0.0394 (0.0344-0.0444) |  | 109778.2 (71377.7-157680.2) | 69.4 (45.3-100.2) | 0.064 (0.0588-0.0693) |
| Barbados | 2116.9 (1875.4-2375) | 801.4 (699.7-918.3) | -0.0068 (-0.0071--0.0065) |  | 3708.9 (3299.9-4147.3) | 1414.1 (1242.3-1605.8) | -0.0067 (-0.007--0.0064) |  | 126 (83-180.7) | 48.3 (31.7-70.5) | -0.011 (-0.0138--0.0082) |
| Belarus | 90420.1 (80395.5-101442.2) | 1099.7 (951.3-1258) | -0.0088 (-0.0099--0.0077) |  | 158447.5 (140837.5-177229.6) | 1936.6 (1681.9-2220.1) | -0.0087 (-0.0098--0.0076) |  | 5389.7 (3513.7-7746.9) | 66.5 (43.2-96.2) | 8e-04 (-0.0031-0.0046) |
| Belgium | 67943.3 (60314.8-75743.7) | 577.8 (509.1-648.6) | 0 (-8e-04-8e-04) |  | 119428.8 (106457.7-132621.3) | 1022.5 (905.3-1143.9) | 5e-04 (-3e-04-0.0013) |  | 3966.7 (2657.3-5626.7) | 34.2 (22.5-48.8) | -0.0048 (-0.0074--0.0022) |
| Belize | 3300.2 (2860.1-3790.8) | 800.2 (698.5-917) | 0.0087 (0.0081-0.0094) |  | 5801.4 (5050.5-6662) | 1412 (1240.4-1604.5) | 0.0086 (0.0079-0.0092) |  | 199 (129.6-288.5) | 48.1 (31.3-69.9) | 0.0076 (0.0044-0.0109) |
| Benin | 117833.6 (99960.8-139148.2) | 802.7 (700.6-919.9) | -0.0044 (-0.0055--0.0033) |  | 210104.2 (177995.7-248647.8) | 1416.4 (1244.3-1609.2) | -0.0046 (-0.0056--0.0035) |  | 7120.3 (4664.3-10503.7) | 48.1 (31.3-69.8) | 0.0115 (0.009-0.014) |
| Bermuda | 440.7 (391.5-491.7) | 801.3 (699.5-918.1) | -0.0046 (-0.0066--0.0026) |  | 771.8 (688.8-856) | 1413.9 (1242.2-1606.1) | -0.0045 (-0.0064--0.0026) |  | 26.1 (17.2-37.1) | 48.3 (31.6-69.5) | -0.0038 (-0.0077-0) |
| Bhutan | 8488.8 (7358.9-9689.2) | 1146.1 (999.2-1309.7) | 0.0024 (-6e-04-0.0054) |  | 14935.3 (13039.1-17034.7) | 2020.3 (1765.5-2300.8) | 0.0021 (-9e-04-0.0051) |  | 508.2 (331.7-737.7) | 68.5 (44.7-99.7) | 0.019 (0.017-0.0209) |
| Bolivia (Plurinational State of) | 100825 (86976-116652.7) | 799.9 (698.3-916.6) | -0.0088 (-0.0097--0.0079) |  | 178798.1 (155153.4-205861.8) | 1411.6 (1240-1603.1) | -0.0087 (-0.0095--0.0078) |  | 6080.6 (3954-8835.8) | 48 (31.3-69.7) | 0.0045 (0.0022-0.0068) |
| Bosnia and Herzegovina | 31731.3 (28243-35725) | 1168.3 (1015.5-1347.3) | -0.011 (-0.0171--0.0049) |  | 55467.4 (49295.6-62269.8) | 2058.3 (1783-2377.3) | -0.0109 (-0.017--0.0048) |  | 1881 (1246.7-2695) | 70.5 (46.2-102) | -0.0022 (-0.0076-0.0031) |
| Botswana | 19101.5 (16516.8-22028.6) | 802.6 (700.9-919.3) | -0.0143 (-0.0151--0.0135) |  | 33661.8 (29322.6-38564.7) | 1416.3 (1244.5-1607.8) | -0.0141 (-0.0148--0.0133) |  | 1145.4 (749.7-1654.3) | 47.9 (31.4-69.1) | -0.0112 (-0.0158--0.0067) |
| Brazil | 1734475.6 (1525201.8-1960549.8) | 848.3 (742.7-970.1) | -0.0011 (-0.0014--8e-04) |  | 3048157.5 (2685291.7-3446746.3) | 1497.2 (1307.5-1709.2) | -0.0011 (-0.0014--8e-04) |  | 103529.6 (67672-149034.3) | 50.9 (33.2-73.7) | 0.009 (0.0069-0.0111) |
| Brunei Darussalam | 3144.8 (2753.2-3575.6) | 749.9 (655.3-853.7) | 0.0103 (-0.0056-0.0261) |  | 5528.6 (4862.9-6280.5) | 1322.9 (1166.8-1501.1) | 0.0094 (-0.0067-0.0254) |  | 189.2 (122.3-275.1) | 45.1 (29.1-65.4) | 0.0179 (0.0021-0.0337) |
| Bulgaria | 66312.7 (59094.2-74464.3) | 1165.4 (1013-1343.7) | -0.0131 (-0.0159--0.0104) |  | 116132.8 (103494.3-130196.6) | 2053.2 (1778.5-2371) | -0.0133 (-0.016--0.0105) |  | 3929.6 (2637.6-5598.3) | 70.5 (46.4-102.8) | -0.0062 (-0.0093--0.003) |
| Burkina Faso | 211647.5 (179844.8-249984.2) | 804.4 (702.3-921.7) | -0.0047 (-0.0054--0.0039) |  | 377929.6 (320590.1-446858) | 1419.4 (1247.1-1612.8) | -0.0046 (-0.0054--0.0039) |  | 12710.9 (8288.3-18628.2) | 48 (31.6-68.8) | 0.0233 (0.0185-0.0281) |
| Burundi | 110712.4 (94050.9-130784.1) | 800.1 (698.3-917) | -0.0237 (-0.0257--0.0216) |  | 197340.4 (167437.9-233222.1) | 1411.9 (1239-1605) | -0.0235 (-0.0256--0.0215) |  | 6678.5 (4325.2-9856.4) | 47.7 (31.1-68.9) | -0.0152 (-0.0232--0.0073) |
| Cabo Verde | 4498.1 (3907.3-5155.7) | 799.8 (698.2-915.8) | -0.0384 (-0.0392--0.0377) |  | 7923.4 (6921.1-9072.3) | 1411.4 (1239.7-1602) | -0.038 (-0.0387--0.0373) |  | 271.3 (174.3-394.2) | 48.1 (30.9-69.6) | -0.0339 (-0.0369--0.0309) |
| Cambodia | 121662.6 (105238.1-139241.9) | 717.6 (627.2-818.3) | 0.0161 (0.0154-0.0168) |  | 215268.7 (187706.9-246698.3) | 1269.7 (1113-1442.9) | 0.0164 (0.0158-0.017) |  | 7331.5 (4723.9-10410.6) | 43 (27.8-61.1) | 0.0461 (0.0415-0.0506) |
| Cameroon | 259415.3 (221598.5-304961.9) | 800.8 (699.2-917.6) | -0.0064 (-0.0067--0.0061) |  | 459426 (393696.6-539118.2) | 1413.2 (1241.5-1605.7) | -0.0063 (-0.0066--0.006) |  | 15680.2 (10183.2-23016.3) | 48 (31.6-69.8) | 0.0175 (0.0119-0.0231) |
| Canada | 296113.8 (261749.8-331572.3) | 924.9 (806.8-1056.2) | -8e-04 (-0.0025-9e-04) |  | 518471.6 (459809.5-580057.6) | 1630.2 (1426.5-1858.6) | -7e-04 (-0.0024-9e-04) |  | 17598.9 (11526.2-25123.8) | 55.7 (36.4-79.6) | -0.0071 (-0.0113--0.003) |
| Central African Republic | 47928.3 (40813.1-56262.5) | 803.7 (701.7-920.9) | 0.0026 (2e-04-0.0049) |  | 85361.6 (72837.9-100155.1) | 1418.2 (1246.1-1611.6) | 0.0025 (2e-04-0.0049) |  | 2875.7 (1878.5-4209.5) | 47.7 (31.3-68.8) | 0.0222 (0.0178-0.0265) |
| Chad | 158735.5 (133747.2-189340.4) | 800.4 (698.6-916.9) | -0.0194 (-0.0201--0.0187) |  | 284472.5 (239368.7-339291.8) | 1412.3 (1240.3-1606.9) | -0.0191 (-0.0198--0.0184) |  | 9585.8 (6208.1-14300.2) | 47.8 (31.4-68.5) | -0.0129 (-0.0164--0.0094) |
| Chile | 129021.8 (114082.7-145194) | 761.2 (665.7-866.1) | -0.0106 (-0.0128--0.0085) |  | 226356.5 (200740.2-253603.1) | 1342.7 (1183.5-1527.2) | -0.0106 (-0.0128--0.0085) |  | 7700.7 (4997-11029.5) | 45.8 (29.4-66.3) | -0.0043 (-0.0081--5e-04) |
| China | 9636079.7 (8483206.1-10771152.1) | 738.7 (645.6-839.9) | -0.01 (-0.0109--0.0091) |  | 16930669.1 (14975677.4-18887307.8) | 1305.7 (1145.2-1491.7) | -0.01 (-0.0109--0.009) |  | 576544.5 (379886.1-818597) | 44.6 (29.2-65) | 0.0031 (0.0014-0.0047) |
| Colombia | 364938.9 (320420.1-412380.3) | 801.2 (699.5-918.1) | -5e-04 (-0.0011-2e-04) |  | 642984.1 (567328.8-726692.1) | 1413.9 (1242.1-1605.2) | -4e-04 (-0.0011-2e-04) |  | 21931.1 (14361.4-31310) | 48.3 (31.6-69.7) | 0.0119 (0.009-0.0147) |
| Comoros | 5912 (5110.2-6837) | 800.5 (698.8-917) | -0.007 (-0.0077--0.0062) |  | 10432.1 (9066-12002.6) | 1412.6 (1240.9-1603.7) | -0.0069 (-0.0076--0.0061) |  | 356.4 (230.4-524.7) | 48 (31-70) | 0.0115 (0.0078-0.0152) |
| Congo | 45730.3 (39269.4-53303.2) | 801.5 (699.6-918.5) | -0.0125 (-0.013--0.012) |  | 80860.4 (69536.9-94079.8) | 1414.4 (1242.1-1607.8) | -0.0122 (-0.0127--0.0117) |  | 2751.1 (1793.7-3983.6) | 47.9 (31.3-68.9) | 0.0045 (3e-04-0.0086) |
| Cook Islands | 120.2 (106.2-136.3) | 697.5 (613.2-794.9) | 0.0216 (0.0189-0.0242) |  | 211.9 (188-240) | 1232.7 (1084.7-1409.5) | 0.021 (0.0185-0.0235) |  | 7.2 (4.6-10.3) | 41.9 (27.3-60.6) | 0.0166 (0.0134-0.0198) |
| Costa Rica | 35769 (31535.2-40457.9) | 802.2 (700.4-919.3) | 0.0118 (0.0107-0.0128) |  | 62832.3 (55393.5-71003.6) | 1415.6 (1243.7-1607.9) | 0.0116 (0.0105-0.0126) |  | 2145.9 (1411.6-3079.1) | 48.3 (31.7-70) | 0.0149 (0.0107-0.019) |
| Croatia | 40595 (36211.3-45653.6) | 1166.8 (1014.2-1345.4) | -0.0141 (-0.0155--0.0126) |  | 71009.1 (63295.7-79516.1) | 2055.8 (1780.6-2374) | -0.0138 (-0.0152--0.0123) |  | 2400.8 (1564.6-3432.3) | 70.5 (45.6-102.6) | -0.0067 (-0.0086--0.0049) |
| Cuba | 80052.1 (70835.6-89651) | 798.4 (697-914.6) | -0.0023 (-0.0023--0.0022) |  | 140236 (125024.7-156440.1) | 1408.9 (1237.6-1599.3) | -0.0022 (-0.0023--0.0021) |  | 4747.3 (3117.8-6825.6) | 48 (31.1-69.4) | 1e-04 (-0.0036-0.0038) |
| Cyprus | 8050.5 (7112.7-9020.4) | 580.1 (510.3-651.6) | 0.0239 (0.0212-0.0267) |  | 14167.8 (12562.2-15967.8) | 1026.4 (908-1148.4) | 0.0233 (0.0206-0.026) |  | 473.9 (312.9-668.9) | 34.5 (22.7-48.8) | 0.0241 (0.0195-0.0287) |
| Czechia | 104183.9 (92611.3-117348.1) | 1164.4 (1011.8-1342.8) | -0.0215 (-0.022--0.0209) |  | 182785.6 (162654.4-205598.5) | 2051.6 (1776.6-2369.5) | -0.0211 (-0.0217--0.0206) |  | 6163.8 (4117.4-8754.2) | 70.3 (46.3-101.8) | -0.0226 (-0.0247--0.0204) |
| C么te d'Ivoire | 233225.1 (198968.4-273460.4) | 796.1 (695.1-912.1) | -0.0054 (-0.0068--0.0039) |  | 414562.3 (354416-486231.7) | 1404.9 (1233.3-1596.4) | -0.0051 (-0.0065--0.0036) |  | 14042.5 (9056.3-20583.1) | 47.5 (31-68.4) | 0.0184 (0.0154-0.0215) |
| Democratic People's Republic of Korea | 174384 (152693.3-197211.7) | 706.9 (618.2-803.5) | -0.0512 (-0.0529--0.0494) |  | 306716 (270323.3-345566.1) | 1249.6 (1094-1429.4) | -0.05 (-0.0517--0.0484) |  | 10446 (6846.9-15008.7) | 42.6 (27.9-61.9) | -0.0482 (-0.0505--0.0459) |
| Democratic Republic of the Congo | 793593.1 (675203.3-933769.5) | 801.2 (699.4-917.7) | -0.0067 (-0.0073--0.006) |  | 1407877.7 (1200985.3-1656248.6) | 1413.8 (1242.1-1605.3) | -0.0066 (-0.0072--0.006) |  | 47686.8 (31258.1-69836.3) | 47.7 (31.3-68.9) | 0.0178 (0.0123-0.0232) |
| Denmark | 39927.6 (35475.9-44419.7) | 683.9 (602.7-766.6) | -0.002 (-0.0038--1e-04) |  | 69623.1 (61919.5-77579.5) | 1197.9 (1058.1-1347) | -0.0017 (-0.0036-2e-04) |  | 2340.8 (1558.2-3337.9) | 40.7 (26.7-57.4) | 0.0024 (-0.0015-0.0062) |
| Djibouti | 10194.9 (8774.9-11823) | 792.2 (691.8-907.5) | -0.0036 (-0.0062--0.001) |  | 18097.8 (15644.1-20931.7) | 1398.2 (1227.9-1589.1) | -0.0032 (-0.0057--7e-04) |  | 615.7 (398-896.9) | 47.6 (31.1-69.5) | 0.0092 (0.004-0.0144) |
| Dominica | 506.1 (447.4-569.5) | 797.6 (696.3-913.8) | -0.0118 (-0.0125--0.0111) |  | 887 (784.5-997.7) | 1407.6 (1235.5-1599.1) | -0.0118 (-0.0124--0.0111) |  | 30.2 (20-43.2) | 48 (31.5-69.8) | -0.0145 (-0.0168--0.0122) |
| Dominican Republic | 87091.9 (75727.9-99687.6) | 799.3 (697.7-915.8) | -0.0159 (-0.0177--0.0141) |  | 153794.1 (134748.6-175091.4) | 1410.4 (1238.9-1601.5) | -0.0157 (-0.0175--0.0139) |  | 5244.9 (3445-7608.9) | 48.1 (31.6-69.8) | -0.013 (-0.016--0.01) |
| Ecuador | 141010.3 (122553.6-161406.3) | 800.2 (698.6-917.1) | -0.0011 (-0.0019--3e-04) |  | 248539.8 (217617.3-283905.8) | 1412.2 (1240.6-1603.9) | -0.0011 (-0.0018--3e-04) |  | 8516 (5515.7-12414.4) | 48.3 (31.2-70.5) | 0.012 (0.0091-0.015) |
| Egypt | 1174048.1 (1010551.1-1343599.3) | 1138.9 (990.2-1292.3) | -0.0079 (-0.0091--0.0067) |  | 2041960.2 (1760324.6-2333132.7) | 1985 (1717.2-2245.6) | -0.008 (-0.0091--0.0068) |  | 71067.7 (46123.2-102752.2) | 68.6 (44.6-98.9) | 0.0093 (0.006-0.0127) |
| El Salvador | 49870.4 (43465.3-56978.1) | 805.7 (703.5-922.9) | 0.011 (0.0095-0.0125) |  | 87735.7 (76931.4-99931.2) | 1421.6 (1249.2-1614.4) | 0.0108 (0.0094-0.0122) |  | 3000.5 (1935.2-4381.1) | 48.4 (31.3-70.6) | 0.0256 (0.0213-0.0298) |
| Equatorial Guinea | 12287.9 (10469.1-14395.8) | 795.2 (694.8-909.9) | -0.0557 (-0.0598--0.0516) |  | 21699.4 (18612.1-25399.5) | 1403.7 (1233.9-1593.8) | -0.0543 (-0.0584--0.0503) |  | 742.3 (486-1085.3) | 47.5 (31-68.7) | -0.0139 (-0.0212--0.0066) |
| Eritrea | 58951.3 (50387.2-69046) | 801.5 (699.8-917.6) | -0.007 (-0.0077--0.0062) |  | 104388.5 (89465.8-121981.1) | 1414.3 (1242.6-1604.9) | -0.0069 (-0.0076--0.0062) |  | 3557.7 (2337.4-5188.1) | 47.9 (31.6-69.5) | 0.028 (0.0258-0.0302) |
| Estonia | 12168.3 (10826.3-13629.9) | 1094.9 (946.8-1252.1) | -0.0284 (-0.0311--0.0257) |  | 21310.1 (19047.5-23751.3) | 1928.3 (1674.4-2210.5) | -0.0281 (-0.0308--0.0255) |  | 722.6 (473.6-1032.5) | 66.2 (43-96.1) | -0.0151 (-0.0189--0.0114) |
| Eswatini | 9768.8 (8398.9-11382.8) | 803.8 (702-920.5) | -0.0156 (-0.0189--0.0123) |  | 17261.6 (14919.2-19991.9) | 1418.4 (1247.4-1610) | -0.0153 (-0.0185--0.012) |  | 587.6 (382.8-858.8) | 47.9 (31.4-69.2) | -0.0388 (-0.043--0.0346) |
| Ethiopia | 1027321.6 (876210.4-1205176.1) | 845.6 (739.9-967.6) | -0.0019 (-0.0037--1e-04) |  | 1824333.9 (1557942.4-2142030.3) | 1492.5 (1302.9-1704.1) | -0.0019 (-0.0036--1e-04) |  | 62126.2 (40281.2-91625.7) | 50.6 (32.9-73.2) | 0.0257 (0.0224-0.0291) |
| Fiji | 6369.5 (5587.3-7258.4) | 696 (611.8-793.5) | 0.0027 (0.0021-0.0032) |  | 11244.9 (9870-12866.9) | 1230.3 (1082.5-1406.1) | 0.0027 (0.0022-0.0032) |  | 382.1 (248-552.2) | 41.6 (27.2-60.2) | 0.002 (-0.0016-0.0056) |
| Finland | 32799.8 (29291.9-36515.5) | 575.3 (506.8-645.6) | -0.0255 (-0.0257--0.0252) |  | 57588.9 (51234.9-64005.8) | 1018 (901-1139) | -0.0247 (-0.0249--0.0245) |  | 1912.8 (1283.4-2685) | 34.1 (22.6-47.9) | -0.0249 (-0.0282--0.0216) |
| France | 513279.3 (458613.4-566652.2) | 765.3 (678-854.1) | 0.0035 (0.0028-0.0041) |  | 888706.5 (794805.9-984334.6) | 1333.6 (1189.2-1481.2) | 0.0036 (0.003-0.0042) |  | 30142.6 (20119.3-42243.2) | 45.5 (29.6-64.3) | 0.0086 (0.006-0.0113) |
| Gabon | 14695.6 (12676-17051.9) | 804.1 (701.9-921.5) | 0.0105 (0.0102-0.0108) |  | 25962.4 (22511.8-29929.1) | 1418.7 (1246.6-1612.6) | 0.0101 (0.0099-0.0104) |  | 881.9 (576.3-1277.7) | 48 (31.5-69.3) | 0.0193 (0.0163-0.0223) |
| Gambia | 20054 (17105.6-23534.9) | 801.8 (699.9-918.8) | 0.0152 (0.0126-0.0179) |  | 35557.2 (30422.6-41741.1) | 1414.8 (1242.9-1608.2) | 0.015 (0.0125-0.0176) |  | 1206.4 (790.7-1750.8) | 47.8 (31.5-69.2) | 0.0231 (0.0202-0.0259) |
| Georgia | 35975.5 (31789.6-40553.6) | 1096.1 (948.3-1254.1) | -0.0255 (-0.0291--0.0219) |  | 63030.6 (55815-70837) | 1930.2 (1676.3-2213.5) | -0.0256 (-0.0293--0.022) |  | 2146.2 (1418.7-3074.6) | 66.1 (43.2-96.3) | -0.0281 (-0.0338--0.0225) |
| Germany | 422220.8 (382465.8-462946.3) | 473.4 (426.9-521.1) | -0.0178 (-0.0222--0.0134) |  | 749373.2 (678696.6-819537.9) | 846.6 (765.5-930.3) | -0.0172 (-0.0217--0.0126) |  | 24603.9 (16476.8-34749.5) | 28.1 (18.6-40) | -0.0211 (-0.0268--0.0154) |
| Ghana | 270409.2 (232725.1-315404.8) | 803.8 (701.9-921) | 0.0129 (0.0114-0.0143) |  | 478394 (413053.6-554626.8) | 1418.4 (1246.2-1611.1) | 0.0127 (0.0113-0.0141) |  | 16314.4 (10556-23756.6) | 48.2 (31.5-69.7) | 0.026 (0.0233-0.0286) |
| Greece | 69452.3 (61627.8-77247) | 659.4 (582.2-737.5) | 0.0011 (-0.0054-0.0077) |  | 121061.6 (107918.2-134677.3) | 1158.1 (1026.4-1293.7) | 0.0013 (-0.0054-0.008) |  | 4055.2 (2684-5712.9) | 39.2 (25.6-55.9) | -0.0069 (-0.0147-8e-04) |
| Greenland | 475 (417.2-533.9) | 914.8 (797.6-1043.8) | 0.0359 (0.0334-0.0384) |  | 834.9 (734.3-942) | 1612.9 (1409.8-1840.1) | 0.0358 (0.0333-0.0382) |  | 28.3 (18.4-40.2) | 54.8 (36-78.4) | 0.0499 (0.0465-0.0533) |
| Grenada | 770.3 (678-869.2) | 797.4 (696.2-913.2) | -0.0231 (-0.0266--0.0196) |  | 1352.5 (1191.4-1527.9) | 1407.2 (1235.5-1598.1) | -0.0228 (-0.0261--0.0195) |  | 46.1 (30-66.4) | 47.9 (31.2-70.2) | -0.0268 (-0.0313--0.0223) |
| Guam | 1166.2 (1026.7-1325.4) | 694.3 (610.1-791.5) | 0.0041 (7e-04-0.0075) |  | 2060.6 (1814.4-2353.1) | 1227.2 (1079.5-1402.6) | 0.0039 (6e-04-0.0072) |  | 70 (46.1-100.6) | 41.7 (27.6-60.1) | -0.001 (-0.0057-0.0037) |
| Guatemala | 148435.5 (128326.4-171875.7) | 803.5 (701.6-920.5) | 0.0106 (0.0091-0.012) |  | 261951.3 (227622.1-301969.3) | 1417.9 (1245.9-1610.5) | 0.0106 (0.0091-0.012) |  | 8969.7 (5826.7-13099.6) | 48.3 (31.5-70.2) | 0.0278 (0.0229-0.0326) |
| Guinea | 117034.7 (99437.1-138245.4) | 803.5 (701.4-920.3) | 0.0038 (0.0022-0.0054) |  | 208297.4 (177102.8-245904.9) | 1417.9 (1245.6-1612.9) | 0.0037 (0.0021-0.0053) |  | 7055.4 (4588.6-10371) | 48 (31.3-69.7) | 0.0123 (0.0088-0.0159) |
| Guinea-Bissau | 17130.3 (14608.4-20146.6) | 804.1 (702-921.3) | -0.001 (-0.0013--7e-04) |  | 30388.8 (25959-35730.2) | 1418.8 (1246.7-1612.3) | -0.001 (-0.0013--7e-04) |  | 1031.1 (669-1486.8) | 48 (31.2-68.7) | 0.0109 (0.0077-0.0141) |
| Guyana | 6115.9 (5325.1-6980.9) | 801.1 (699.4-918.1) | -0.0014 (-0.004-0.0012) |  | 10769.7 (9421.6-12303.7) | 1413.7 (1242.1-1606) | -0.0013 (-0.0038-0.0013) |  | 365.9 (239-531.9) | 47.8 (31.2-69.7) | 0.0079 (0.0046-0.0112) |
| Haiti | 105348.7 (90729.8-122507.6) | 803.4 (701.4-920.5) | -6e-04 (-0.0012-1e-04) |  | 186462 (161385.6-215551.5) | 1417.7 (1245.6-1611.3) | -4e-04 (-0.001-3e-04) |  | 6319.6 (4174.8-9131.6) | 47.9 (31.7-68.7) | -0.0031 (-0.0071-9e-04) |
| Honduras | 82399.7 (71137.4-95513.3) | 802.8 (700.9-919.9) | 0.0087 (0.0077-0.0096) |  | 145474.6 (126054.9-167763) | 1416.6 (1244.6-1609.7) | 0.0086 (0.0076-0.0095) |  | 4984.9 (3236-7334.6) | 48.3 (31.4-70.7) | 0.0164 (0.0137-0.0192) |
| Hungary | 92973.7 (82843.5-104567.4) | 1169.4 (1016.8-1348.4) | -0.0131 (-0.0152--0.0111) |  | 162720.9 (145056.1-182826.5) | 2060.2 (1785.2-2378.9) | -0.0131 (-0.0151--0.0111) |  | 5503.2 (3658.8-7861.1) | 70.6 (46.4-102.8) | -0.0064 (-0.0105--0.0022) |
| Iceland | 2026.7 (1797.4-2258.1) | 573.5 (505.3-643.8) | -0.0158 (-0.0235--0.0081) |  | 3568.7 (3173.1-3994.1) | 1015 (898-1135.8) | -0.0157 (-0.0233--0.008) |  | 119.4 (78.7-168) | 34.1 (22.3-47.8) | -0.0134 (-0.0229--0.0039) |
| India | 12552793 (10916084.7-14379341.2) | 919.3 (799.2-1057.3) | 0.0083 (0.0075-0.009) |  | 22065409.4 (19265922-25287088.5) | 1623.2 (1420.4-1860.8) | 0.0079 (0.0072-0.0086) |  | 749495.9 (490301.3-1074876) | 54.8 (35.8-78.9) | 0.0303 (0.0288-0.0319) |
| Indonesia | 1951062.2 (1701878.6-2218261.2) | 758.3 (663.5-861.2) | 0.0045 (0.0043-0.0047) |  | 3436330.5 (2999536.2-3920261.6) | 1341.7 (1174.2-1528.9) | 0.0045 (0.0043-0.0047) |  | 117356.8 (76971.2-167280.4) | 45.5 (30.1-65.1) | 0.0225 (0.0205-0.0245) |
| Iran (Islamic Republic of) | 796507.8 (701287.1-903790.2) | 969.8 (846.4-1100.5) | 0.0014 (-7e-04-0.0035) |  | 1401912.6 (1239909-1585599.8) | 1711.7 (1502.8-1946.2) | 0.0015 (-6e-04-0.0036) |  | 47757.1 (31138.8-68212.3) | 58.2 (38.3-84.1) | 0.0075 (0.0051-0.0098) |
| Iraq | 399652.2 (342509.7-462130.7) | 914.9 (799.5-1044.5) | 0.0013 (-4e-04-0.0031) |  | 704960.8 (610445-816672.6) | 1613.9 (1415.5-1842.9) | 0.0013 (-5e-04-0.003) |  | 24121 (15681.3-35222) | 54.8 (35.6-79) | 0.0154 (0.0122-0.0186) |
| Ireland | 29225.6 (25916.4-32646.3) | 580.2 (511.1-651.5) | 0.0083 (0.0015-0.0152) |  | 51425.2 (45623.1-57462.4) | 1026.8 (908.8-1148.2) | 0.0087 (0.0019-0.0156) |  | 1718.3 (1135.8-2397.7) | 34.4 (22.4-48.9) | 0.006 (-0.0019-0.0139) |
| Israel | 48247.9 (43270.2-53851.9) | 517 (462.7-577.6) | -0.0259 (-0.0269--0.025) |  | 85853.8 (76725.5-95596.7) | 920.7 (821.7-1025.8) | -0.0255 (-0.0264--0.0247) |  | 2864.2 (1901-4049.5) | 30.8 (20.4-43.5) | -0.0183 (-0.021--0.0155) |
| Italy | 323926 (287413.7-363375.8) | 495 (435.2-556.1) | -0.0178 (-0.0199--0.0158) |  | 569033.1 (504991.1-641093.6) | 878.1 (772.4-987.2) | -0.0176 (-0.0196--0.0156) |  | 18837.6 (12521.3-26446.4) | 29.3 (19.4-41.5) | 9e-04 (-0.0022-0.0041) |
| Jamaica | 21090.9 (18569-23810.6) | 800.2 (698.6-917.1) | -0.0079 (-0.0083--0.0074) |  | 36977.5 (32531.9-41878.4) | 1412.1 (1240.7-1604.6) | -0.0077 (-0.0082--0.0073) |  | 1263.8 (833.5-1811.7) | 48.1 (31.6-69.7) | -0.0134 (-0.0159--0.0109) |
| Japan | 867514.3 (773290.3-966755.7) | 802.8 (703.5-919.3) | -0.0121 (-0.0127--0.0116) |  | 1517154.4 (1357988.2-1687941.2) | 1416.7 (1243.7-1619.8) | -0.0119 (-0.0125--0.0113) |  | 51222.4 (34088.3-72624.2) | 48.5 (31.8-69.6) | -0.0043 (-0.0052--0.0034) |
| Jordan | 108564 (93312.3-125305.9) | 908.6 (794-1037.7) | -0.0127 (-0.0184--0.0069) |  | 191186.7 (165869.1-220492.8) | 1603 (1404.6-1830.2) | -0.0124 (-0.018--0.0067) |  | 6578.7 (4263.2-9588.3) | 54.8 (35.4-79.7) | -0.006 (-0.013-9e-04) |
| Kazakhstan | 201404.5 (174543.4-231488.7) | 1100.9 (952.5-1259.6) | -0.0038 (-0.0057--0.0019) |  | 354331.3 (308042.7-405427.5) | 1938.8 (1683.8-2223.6) | -0.0037 (-0.0056--0.0018) |  | 12147.3 (7961.4-17512) | 66.4 (43.3-96.2) | 0.0056 (0.0029-0.0082) |
| Kenya | 460818.1 (395274.5-536435.2) | 847.7 (741.9-969.6) | -0.0025 (-0.0035--0.0016) |  | 814013.3 (700041.5-943996.5) | 1496.1 (1306.2-1708) | -0.0025 (-0.0035--0.0016) |  | 27899.1 (18158.1-40710.2) | 50.9 (33.2-73.7) | 0.0158 (0.0122-0.0194) |
| Kiribati | 866.6 (751.5-995.4) | 698.4 (614.1-796.1) | 0.0016 (6e-04-0.0025) |  | 1535.1 (1334-1780.6) | 1234.4 (1086.3-1410.8) | 0.0015 (6e-04-0.0025) |  | 52.1 (34.2-75.6) | 41.7 (27.5-60) | 0.0036 (1e-04-0.0071) |
| Kuwait | 38020.2 (32963.2-43449.4) | 907.9 (792.6-1037.9) | 0.0404 (0.0253-0.0556) |  | 66872.6 (58563.3-75817.5) | 1601.8 (1401.8-1829) | 0.0399 (0.0251-0.0547) |  | 2279.8 (1492.7-3284.7) | 54.6 (35.8-79.1) | 0.0351 (0.0188-0.0513) |
| Kyrgyzstan | 75380.4 (64687.2-87281.2) | 1098.4 (950.1-1256.6) | -0.0052 (-0.0067--0.0037) |  | 132586.8 (113839.8-153609.1) | 1934.5 (1680.2-2218.9) | -0.0051 (-0.0066--0.0036) |  | 4570 (2965-6639) | 66.3 (43-96) | 0.0051 (0.0013-0.0089) |
| Lao People's Democratic Republic | 53076.3 (45830.3-60959.3) | 718.8 (628.1-820) | 0.007 (0.0065-0.0075) |  | 93948 (81682.8-107919.9) | 1271.7 (1115-1444.3) | 0.0071 (0.0066-0.0076) |  | 3206 (2094.8-4562.5) | 43.2 (28.3-61.5) | 0.0225 (0.0188-0.0261) |
| Latvia | 17814.9 (15861.7-19884.3) | 1098.1 (949.8-1256.1) | -0.0181 (-0.0204--0.0157) |  | 31220.3 (27896.8-34718.6) | 1933.8 (1679.5-2217.3) | -0.0179 (-0.0203--0.0156) |  | 1055.4 (705.4-1493.2) | 66.3 (43.5-95.6) | -0.0064 (-0.0106--0.0021) |
| Lebanon | 47214.5 (41231.4-54030.6) | 917.5 (801.9-1046.6) | 0.0053 (0.0032-0.0075) |  | 83385 (73488.3-94823.5) | 1618.7 (1420.9-1845.9) | 0.0054 (0.0033-0.0075) |  | 2830.6 (1838.3-4098.5) | 55.1 (35.9-79.9) | 0.0117 (0.008-0.0155) |
| Lesotho | 17223 (14895.6-19892.9) | 802.8 (701-919) | -0.0021 (-0.0042-0) |  | 30373.7 (26388.9-34962.1) | 1416.7 (1244.8-1607.4) | -0.0021 (-0.0042-0) |  | 1031.9 (676-1523.7) | 47.8 (31.2-69.4) | -0.0218 (-0.0239--0.0198) |
| Liberia | 41738.8 (35755.7-48896.8) | 798.8 (697.3-915.3) | -0.0134 (-0.0155--0.0112) |  | 73776.3 (63248.9-86147.3) | 1409.7 (1237.6-1602.6) | -0.0132 (-0.0153--0.011) |  | 2507.4 (1634-3638.7) | 47.5 (31-68.3) | 0.0201 (0.0147-0.0255) |
| Libya | 58105.4 (50708.6-66436.5) | 913.5 (798.1-1043.2) | -0.0078 (-0.0136--0.002) |  | 101798.8 (89479.2-115675.6) | 1611.5 (1413.2-1840.4) | -0.0074 (-0.013--0.0018) |  | 3478.3 (2259.3-4961.7) | 54.8 (35.5-79.4) | -0.0141 (-0.0205--0.0076) |
| Lithuania | 25737.3 (22951.4-28680.7) | 1098.8 (950.4-1256.9) | -0.0136 (-0.0153--0.012) |  | 45084.4 (40272.9-50074.3) | 1935 (1680.4-2218.4) | -0.0136 (-0.0152--0.0119) |  | 1526.8 (1001.1-2175.8) | 66.5 (43.3-96.5) | -0.0067 (-0.0097--0.0036) |
| Luxembourg | 3714 (3299.5-4144.5) | 574.9 (506.4-645.4) | -0.0202 (-0.0233--0.0172) |  | 6532.9 (5786-7320.1) | 1017.4 (900.5-1138.3) | -0.0196 (-0.0227--0.0166) |  | 217.8 (142.3-303.9) | 34.1 (21.9-47.6) | -0.0123 (-0.0164--0.0083) |
| Madagascar | 237809.5 (202763.1-278975.7) | 800.5 (698.8-917.4) | 4e-04 (-3e-04-0.0012) |  | 421796.6 (360862.1-494562.3) | 1412.5 (1240.9-1604.6) | 5e-04 (-3e-04-0.0012) |  | 14394.6 (9421.3-20840.7) | 48 (31.5-69.7) | 0.03 (0.0266-0.0334) |
| Malawi | 165456.1 (141163.5-194871.6) | 804.3 (702.2-921.5) | 0.0075 (0.0065-0.0086) |  | 292297 (249732.9-342917.7) | 1419.2 (1247-1612.8) | 0.0075 (0.0064-0.0085) |  | 9977.9 (6489.4-14632.4) | 48 (31.3-70) | 0.0333 (0.0286-0.038) |
| Malaysia | 225736.3 (196659.4-256844.1) | 720.1 (629.1-821.9) | 0.005 (0.0044-0.0055) |  | 397836.6 (348398.1-453344.6) | 1274 (1117.2-1446.4) | 0.005 (0.0044-0.0055) |  | 13568 (8793.8-19420.4) | 43.2 (28.3-62.2) | 0.0146 (0.0115-0.0177) |
| Maldives | 3564.1 (3099-4060.2) | 727.6 (635.2-830.6) | 0.034 (0.0234-0.0446) |  | 6269.3 (5462.8-7152.7) | 1287.2 (1128.8-1462.2) | 0.0351 (0.0245-0.0457) |  | 215.3 (139.8-310) | 43.8 (28.7-63.4) | 0.0645 (0.054-0.0749) |
| Mali | 206135.4 (174429.1-243943) | 800.7 (699-917.4) | -0.0076 (-0.0092--0.0061) |  | 368685.3 (311088.7-438304.8) | 1412.9 (1241.1-1607) | -0.0074 (-0.009--0.0059) |  | 12394.6 (8135.2-18231) | 47.7 (31.4-69.4) | 0.0145 (0.0094-0.0196) |
| Malta | 2635.8 (2345.5-2932.5) | 574 (505.7-644.3) | -0.0405 (-0.0418--0.0392) |  | 4630.2 (4124.6-5169.5) | 1015.9 (899-1136.6) | -0.0397 (-0.041--0.0384) |  | 154 (101.3-218.7) | 34.1 (22.5-48.3) | -0.0428 (-0.0456--0.04) |
| Marshall Islands | 406.8 (354.5-467.2) | 694.5 (610.3-791.7) | -0.0076 (-0.0086--0.0066) |  | 719 (627.4-829.9) | 1227.6 (1079.7-1404) | -0.0075 (-0.0085--0.0065) |  | 24.5 (16-35.4) | 41.5 (27.2-59.5) | -0.0088 (-0.0115--0.0061) |
| Mauritania | 35262.3 (30231.3-41339.2) | 801.8 (699.8-918.9) | 0.0023 (0.0018-0.0029) |  | 62225.9 (53499.8-72711.8) | 1414.8 (1242.9-1607.9) | 0.0023 (0.0017-0.0028) |  | 2126.3 (1393.3-3087) | 48 (31.4-68.4) | 0.0076 (0.0048-0.0105) |
| Mauritius | 8700.9 (7636.8-9788) | 719.1 (628.5-820.4) | 0.0012 (9e-04-0.0014) |  | 15282.6 (13513.1-17195.3) | 1272.3 (1115.6-1445.1) | 0.0011 (8e-04-0.0013) |  | 515.9 (337.1-731.4) | 43 (27.9-61.4) | 0.0025 (-0.001-0.0059) |
| Mexico | 1034124.2 (905531.1-1177886.3) | 848.6 (742.8-970.7) | -9e-04 (-0.0021-4e-04) |  | 1818867.8 (1596589.7-2071118.5) | 1497.7 (1308.1-1709.8) | -0.001 (-0.0022-3e-04) |  | 62153.5 (40536-90248.9) | 51 (33.3-74.2) | -0.0015 (-0.0045-0.0016) |
| Micronesia (Federated States of) | 726.2 (630.8-834.7) | 695.7 (611.5-793.1) | 0.0025 (0.0012-0.0038) |  | 1280.9 (1120.1-1481.5) | 1229.8 (1082.2-1405.3) | 0.0025 (0.0012-0.0038) |  | 43.8 (28.2-63.8) | 41.7 (27.1-60.4) | 0.008 (0.0051-0.0108) |
| Monaco | 225.3 (200.5-250.5) | 580.6 (511.2-652.2) | 0.0064 (-0.0028-0.0156) |  | 395.3 (351.8-438.8) | 1027.4 (909.4-1149.2) | 0.0069 (-0.0023-0.0161) |  | 13.1 (8.8-18.6) | 34.5 (22.3-48.7) | 0.0041 (-0.0057-0.014) |
| Mongolia | 38407.3 (33003.5-44533.9) | 1098.7 (950.4-1256.8) | 0.012 (0.0107-0.0134) |  | 67921.5 (58547.5-78558.5) | 1935 (1680.5-2219.1) | 0.0118 (0.0104-0.0131) |  | 2328.3 (1506.8-3370.4) | 66.4 (43.2-96.1) | 0.0276 (0.0253-0.0298) |
| Montenegro | 6220.8 (5528.5-7036.4) | 1166.3 (1013.9-1344.9) | -0.0067 (-0.0086--0.0049) |  | 10897 (9665.4-12300.4) | 2054.8 (1780.3-2372.9) | -0.0067 (-0.0085--0.0048) |  | 371.2 (241.8-535.3) | 70.5 (45.6-102.9) | -0.0065 (-0.0098--0.0032) |
| Morocco | 323348.4 (281863.4-368247.3) | 916.9 (801.1-1047) | -0.0112 (-0.0141--0.0083) |  | 568411.8 (499401.7-648623.6) | 1617.4 (1419-1846.8) | -0.0109 (-0.0138--0.008) |  | 19390.6 (12762-28056.9) | 55 (36.2-79.8) | -0.0095 (-0.0129--0.0061) |
| Mozambique | 276624.3 (234518.7-327404.5) | 805.7 (703.4-923.2) | -2e-04 (-0.0014-0.0011) |  | 491890.6 (416205.1-582055.8) | 1421.7 (1249.1-1615.3) | -3e-04 (-0.0015-9e-04) |  | 16687.3 (10946.3-24381.3) | 48 (31.8-69.8) | 0.0213 (0.0171-0.0255) |
| Myanmar | 392898.6 (342057.1-448127.3) | 716.8 (626.6-817.4) | -0.0073 (-0.0075--0.0071) |  | 693963.5 (606077.7-790389.6) | 1268.3 (1111.8-1441.4) | -0.007 (-0.0072--0.0068) |  | 23582.8 (15384.6-33704.2) | 42.9 (28.1-61.4) | 0.0122 (0.0083-0.0162) |
| Namibia | 20471.6 (17630.8-23840.4) | 804.5 (702.4-921.6) | 0.0034 (0.0027-0.0041) |  | 36207.2 (31323.1-41908.6) | 1419.6 (1247.5-1612.2) | 0.0035 (0.0028-0.0041) |  | 1232.7 (813.6-1801.5) | 48.1 (31.9-70) | 0.0066 (0.0027-0.0104) |
| Nauru | 78 (67.4-90.2) | 696.3 (612-793.2) | 0.0057 (-1e-04-0.0115) |  | 138.5 (119.6-161.6) | 1230.8 (1082.9-1406.2) | 0.0066 (7e-04-0.0124) |  | 4.7 (3.1-6.9) | 41.8 (27.5-60.2) | 0.0044 (-0.0015-0.0103) |
| Nepal | 474857.2 (437555.7-515897.9) | 1534.6 (1416.2-1662.5) | 0.0408 (0.0359-0.0458) |  | 823241.5 (752571.9-900552) | 2665.5 (2445.1-2904.2) | 0.0396 (0.0347-0.0445) |  | 28577.7 (18702.8-40449.5) | 92 (60.3-130.2) | 0.0634 (0.0577-0.0691) |
| Netherlands | 102137.1 (90694.2-113727.5) | 577.2 (508.4-648) | 0.0021 (9e-04-0.0034) |  | 179618.5 (159724.9-199737.3) | 1021.4 (904.1-1142.9) | 0.0025 (0.0012-0.0038) |  | 5994.3 (3952.8-8457.2) | 34.4 (22.7-48.1) | 0.0026 (-0.0013-0.0065) |
| New Zealand | 37565.1 (33287-42128.6) | 899.1 (784.2-1022.7) | 0.0173 (0.0144-0.0202) |  | 66017 (58686.1-73851.1) | 1587 (1394.6-1797.4) | 0.0171 (0.0142-0.0199) |  | 2218.7 (1461.8-3177.1) | 53.7 (34.9-77.2) | 0.027 (0.0214-0.0325) |
| Nicaragua | 53189.1 (46094.5-61356.6) | 801.6 (699.9-918.4) | -0.0052 (-0.0057--0.0046) |  | 93656.4 (81417.2-107511.2) | 1414.5 (1242.7-1605.8) | -0.005 (-0.0055--0.0044) |  | 3223 (2105.5-4676.6) | 48.4 (31.6-70.1) | 0.015 (0.0115-0.0186) |
| Niger | 227049.8 (190937.8-271401.2) | 801.6 (699.6-918.8) | 0.0074 (0.0057-0.0092) |  | 407291.2 (342523.7-486655.6) | 1414.5 (1242.5-1606.5) | 0.0075 (0.0058-0.0091) |  | 13727.1 (8891.1-20157.2) | 48 (31.3-69.1) | 0.0268 (0.023-0.0306) |
| Nigeria | 2069798.5 (1765779.8-2434824.6) | 852 (745.6-974.8) | 0.0285 (0.0205-0.0364) |  | 3676207.4 (3137587.9-4316526.5) | 1503.5 (1313.5-1716.5) | 0.0284 (0.0206-0.0361) |  | 124536.8 (81400.4-181680.7) | 50.8 (33.4-73.3) | 0.0454 (0.0342-0.0566) |
| Niue | 11.2 (9.9-12.7) | 695.9 (611.6-792.9) | 0.0058 (0.0032-0.0083) |  | 19.8 (17.5-22.4) | 1230 (1082.8-1405.2) | 0.0056 (0.003-0.0081) |  | 0.7 (0.4-1) | 41.7 (26.8-60.6) | 0.0021 (-0.0019-0.0061) |
| North Macedonia | 21016.6 (18619.6-23751.8) | 1160.9 (1008.5-1338.7) | -0.0186 (-0.0211--0.0161) |  | 36847.1 (32648.3-41475.6) | 2045.5 (1770.9-2362.5) | -0.0185 (-0.021--0.0161) |  | 1252.9 (827.2-1796.5) | 70.1 (46-101) | -0.014 (-0.0171--0.0108) |
| Northern Mariana Islands | 273.4 (240.2-311) | 694.4 (610.1-791.7) | -0.0062 (-0.015-0.0026) |  | 481 (424.8-548.3) | 1227.5 (1080-1403.7) | -0.0054 (-0.0139-0.0031) |  | 16.3 (10.6-23.5) | 41.7 (27.6-61.1) | -0.0139 (-0.0231--0.0048) |
| Norway | 33415.3 (29801.4-37380.9) | 607.8 (537.2-681.2) | -0.0189 (-0.0227--0.0152) |  | 58830.6 (52338.6-65793.1) | 1077.1 (954-1211.2) | -0.0184 (-0.0222--0.0147) |  | 1958.4 (1298.4-2765.9) | 36.1 (23.6-51.2) | -0.0092 (-0.0147--0.0036) |
| Oman | 39562.9 (34025.2-45923.2) | 890.1 (778.5-1015.9) | -0.0339 (-0.055--0.0129) |  | 69818 (60840.1-79979.4) | 1571.1 (1374.1-1793.1) | -0.033 (-0.0535--0.0124) |  | 2390.7 (1535.5-3397) | 53.6 (35-77.6) | -0.0232 (-0.0453--0.0011) |
| Pakistan | 2991952 (2556193.8-3484428) | 1213.5 (1057.1-1387.7) | 0.0168 (0.0163-0.0172) |  | 5296112.7 (4533809.6-6165638.7) | 2140.2 (1869.4-2443.5) | 0.0163 (0.0159-0.0168) |  | 180580.9 (117789.7-263500) | 72.6 (47.4-105.1) | 0.023 (0.0211-0.0249) |
| Palau | 115 (101.7-129.7) | 691.4 (607.3-788.4) | -0.018 (-0.0235--0.0126) |  | 202.1 (179.7-227.9) | 1222.5 (1074.9-1397.1) | -0.0172 (-0.0224--0.012) |  | 6.8 (4.5-9.7) | 41.5 (27.2-60.3) | -0.0216 (-0.0274--0.0158) |
| Palestine | 48646 (41399.6-56666.5) | 915.9 (800.4-1045.7) | -0.0078 (-0.0095--0.0061) |  | 85815.8 (73637.7-99482.6) | 1615.7 (1416.8-1844.7) | -0.0078 (-0.0094--0.0062) |  | 2945.9 (1922.7-4340.7) | 54.9 (35.9-79.6) | -0.0102 (-0.0139--0.0066) |
| Panama | 32902.1 (28754.7-37618.1) | 798.8 (697.3-915.3) | 0.0023 (0.0016-0.0029) |  | 57921.6 (50914.3-65605.6) | 1409.6 (1238.3-1600.8) | 0.0022 (0.0016-0.0029) |  | 1983.2 (1289.9-2884.9) | 48.2 (31.3-70.1) | 0.0048 (0.0022-0.0074) |
| Papua New Guinea | 72710 (62850.3-84166.2) | 693.4 (609.3-790.3) | -0.0029 (-0.0034--0.0024) |  | 129520.7 (111917.2-150705.2) | 1225.7 (1078.9-1401.7) | -0.0031 (-0.0035--0.0026) |  | 4375.6 (2865.3-6462.9) | 41.3 (27.1-59.9) | 0.003 (3e-04-0.0057) |
| Paraguay | 55199.9 (47994.5-63339.7) | 798.4 (697-914.7) | -0.0053 (-0.0054--0.0051) |  | 97100.2 (84923-110972.7) | 1409 (1237.6-1599.5) | -0.0052 (-0.0054--0.005) |  | 3322.4 (2146.7-4852.4) | 48 (31.1-69.9) | -0.0078 (-0.0108--0.0048) |
| Peru | 267957 (234047-306032.8) | 799.2 (697.8-915.7) | -0.0099 (-0.011--0.0087) |  | 472246.6 (414863.1-535959.8) | 1410.3 (1238.9-1601.7) | -0.0095 (-0.0106--0.0084) |  | 16126.9 (10445.1-23241.1) | 48.1 (31.2-69.5) | 0.0067 (0.0029-0.0105) |
| Philippines | 870965.3 (755469.7-995655.4) | 758.6 (663.8-861.6) | 0.0031 (0.0028-0.0034) |  | 1543065.1 (1342307.3-1772702.1) | 1342.3 (1174.7-1529.7) | 0.0033 (0.003-0.0036) |  | 52539.4 (34481.8-75675.5) | 45.5 (30-65.6) | 0.0221 (0.0201-0.0242) |
| Poland | 403451.3 (375425.2-434001.5) | 1268.3 (1157.9-1388.5) | -0.0367 (-0.0552--0.0182) |  | 707810.7 (654973.4-763650.5) | 2238.3 (2031.9-2462.5) | -0.035 (-0.0526--0.0174) |  | 23993.6 (15858.5-33798.7) | 76.7 (50.6-110.5) | -0.0195 (-0.0368--0.0023) |
| Portugal | 49786.2 (44090-55799.4) | 456.4 (406.2-512.2) | -0.001 (-0.0053-0.0033) |  | 88602.2 (78871.8-98779.7) | 818.2 (728.6-917.3) | -9e-04 (-0.0052-0.0034) |  | 2901.9 (1933-4090.3) | 27.1 (17.7-38.1) | 0.0065 (-1e-04-0.0131) |
| Puerto Rico | 24400.8 (21687.8-27304.2) | 801.8 (700.1-918.8) | -0.0041 (-0.0045--0.0036) |  | 42630.2 (37998.6-47562.1) | 1414.9 (1243-1606.7) | -0.004 (-0.0045--0.0036) |  | 1442.3 (952.8-2072) | 48.2 (31.5-70.2) | -0.0043 (-0.0073--0.0014) |
| Qatar | 22196.4 (19077.2-25616.1) | 866.4 (755.9-991.5) | -0.0795 (-0.0988--0.0603) |  | 38989.8 (33746.2-44534.2) | 1530.2 (1337-1744) | -0.0774 (-0.0961--0.0587) |  | 1335 (860.7-1887.5) | 52.1 (34.1-75.5) | -0.0719 (-0.0906--0.0532) |
| Republic of Korea | 350725.6 (309937.5-392025.7) | 752.2 (657.2-856.5) | -0.033 (-0.0374--0.0286) |  | 613810.4 (547341.6-682888) | 1327 (1170.3-1507.1) | -0.0325 (-0.0367--0.0283) |  | 20866.7 (13567.6-29611.3) | 45.4 (29.2-65.8) | -0.0177 (-0.0241--0.0113) |
| Republic of Moldova | 33673 (29925.3-37685.3) | 1097.8 (949.5-1255.5) | -0.0195 (-0.0201--0.0189) |  | 58854.3 (52514-65486.9) | 1933.4 (1679.6-2216.3) | -0.0195 (-0.0201--0.0189) |  | 2004.6 (1307.6-2874.6) | 66.4 (43-96.9) | -0.0028 (-0.0047--9e-04) |
| Romania | 177489 (158387.5-196773.6) | 1100.4 (963.4-1241.7) | -0.0141 (-0.0162--0.0119) |  | 311432.9 (279730-346101.3) | 1944.3 (1712.5-2208.8) | -0.014 (-0.0161--0.0118) |  | 10545.2 (6938-14840.8) | 66.5 (43.6-95.6) | -0.0069 (-0.0101--0.0036) |
| Russian Federation | 1502664.1 (1329310.3-1691086) | 1166.1 (1010.2-1340.5) | -0.0056 (-0.0071--0.0041) |  | 2633630 (2336444-2968348) | 2053.9 (1778.9-2370.8) | -0.0054 (-0.0069--0.0039) |  | 89491 (58486.1-128205.3) | 70.4 (46.1-102.6) | 0.0047 (0.003-0.0064) |
| Rwanda | 110445.5 (94606.5-129124) | 804.6 (702.4-921.7) | -3e-04 (-0.0058-0.0052) |  | 195288.9 (168084.5-227403.5) | 1419.7 (1247.5-1611.7) | -3e-04 (-0.0057-0.0052) |  | 6672.7 (4322.3-9789.1) | 48.2 (31.1-69.7) | 0.0341 (0.0255-0.0427) |
| Saint Kitts and Nevis | 437.9 (386.2-492.6) | 799.8 (698.2-916.4) | -0.0103 (-0.0111--0.0094) |  | 767.9 (677.2-864.1) | 1411.3 (1239.5-1603) | -0.0102 (-0.0111--0.0094) |  | 26.2 (17-37.2) | 48.1 (31.4-69.8) | -0.0065 (-0.009--0.004) |
| Saint Lucia | 1263.2 (1115.6-1422.3) | 799.8 (698.3-916.5) | -0.0169 (-0.0175--0.0163) |  | 2212.2 (1958.6-2487.9) | 1411.5 (1240-1603.1) | -0.0166 (-0.0172--0.016) |  | 75.1 (49.6-107.5) | 48 (31.3-69.4) | -0.0165 (-0.0192--0.0137) |
| Saint Vincent and the Grenadines | 846.3 (746.1-955.4) | 797.9 (696.5-914.4) | -0.0128 (-0.0141--0.0116) |  | 1485.2 (1312.2-1674.5) | 1408.1 (1236-1600.2) | -0.0126 (-0.0138--0.0114) |  | 50.5 (33-72.4) | 47.9 (31.4-69.4) | -0.0163 (-0.0192--0.0134) |
| Samoa | 1533.2 (1327.2-1777.2) | 694.8 (610.7-792.1) | 0.0079 (0.0077-0.008) |  | 2699.5 (2343.4-3138.5) | 1228.2 (1080.6-1404.1) | 0.0075 (0.0073-0.0077) |  | 92.6 (60.7-134.8) | 41.7 (27.4-60) | 0.0044 (9e-04-0.0078) |
| San Marino | 199.5 (177.5-221.9) | 586.3 (516.1-658.7) | 0.0625 (0.0575-0.0674) |  | 350.6 (312.4-391.9) | 1037.4 (917-1162.1) | 0.0612 (0.0561-0.0663) |  | 11.7 (7.7-16.7) | 34.8 (22.4-49.4) | 0.0583 (0.0524-0.0642) |
| Sao Tome and Principe | 1740.8 (1498.4-2027.4) | 800 (698.3-916.7) | -0.0104 (-0.012--0.0088) |  | 3063.9 (2642.2-3554.3) | 1411.8 (1239.8-1603.5) | -0.0104 (-0.0119--0.0088) |  | 105.3 (68.7-154.5) | 48.1 (31.5-69.8) | -0.0039 (-0.0067--0.001) |
| Saudi Arabia | 301899.6 (261410.6-345550.1) | 896.4 (783.1-1023.8) | -0.0172 (-0.0262--0.0083) |  | 530296.1 (464093.1-604025.8) | 1582 (1384.5-1806) | -0.0168 (-0.0254--0.0082) |  | 18153.6 (11627.4-26359.3) | 53.9 (35.1-79.2) | -0.0196 (-0.0282--0.011) |
| Senegal | 133588.2 (114188.1-156390.8) | 800.3 (698.8-917) | -0.0132 (-0.0137--0.0127) |  | 236695.8 (203156.7-276750.4) | 1412.4 (1240.8-1604.5) | -0.0127 (-0.0132--0.0122) |  | 8048.4 (5158.7-11815.7) | 47.8 (30.8-69.3) | 0.0031 (-2e-04-0.0064) |
| Serbia | 86529.7 (77085.8-97747.5) | 1164.4 (1012.2-1342.5) | -0.0195 (-0.0229--0.016) |  | 151252.7 (134499.7-170354.8) | 2051.5 (1777.3-2368.5) | -0.0201 (-0.0235--0.0167) |  | 5144.9 (3426.6-7366.2) | 70.4 (46.3-102.4) | -0.0135 (-0.0174--0.0095) |
| Seychelles | 705.5 (617.4-797.9) | 722.2 (630.9-824.3) | 0.0136 (0.0116-0.0156) |  | 1243 (1093.2-1399.4) | 1277.8 (1120.4-1450.4) | 0.0135 (0.0115-0.0155) |  | 42.2 (27.9-59.9) | 43.4 (28.6-62) | 0.0062 (0.0022-0.0102) |
| Sierra Leone | 73738 (62879.2-86414.3) | 800.9 (699-917.9) | -0.0127 (-0.0157--0.0096) |  | 131005.2 (112079.1-153403.9) | 1413.2 (1240.9-1606.7) | -0.0125 (-0.0155--0.0094) |  | 4442.2 (2889.2-6587.4) | 47.9 (31.4-70.3) | 0.007 (0.0031-0.0109) |
| Singapore | 38254.3 (33816.9-43179.3) | 755.2 (660.7-859.7) | -0.0294 (-0.0407--0.0182) |  | 67160.7 (59451.1-75257.6) | 1332.4 (1174.4-1515.5) | -0.028 (-0.0391--0.0169) |  | 2291.7 (1493.9-3275.6) | 45.8 (29.5-66.2) | -0.0127 (-0.0231--0.0023) |
| Slovakia | 53192.3 (47257.8-60079) | 1167 (1014.4-1345.6) | -0.0177 (-0.0195--0.016) |  | 93321.8 (82913.8-105138.8) | 2056.1 (1780.9-2374.4) | -0.0177 (-0.0194--0.016) |  | 3163.2 (2072-4472.8) | 70.5 (45.9-102.4) | -0.0129 (-0.0161--0.0097) |
| Slovenia | 19931.6 (17758.3-22390.5) | 1161.5 (1009-1339.2) | -0.0326 (-0.0339--0.0313) |  | 34898.8 (31082-39244.4) | 2046.5 (1771.7-2364.1) | -0.032 (-0.0333--0.0307) |  | 1180.6 (777.7-1688.4) | 70.2 (46-102.1) | -0.0264 (-0.0299--0.0229) |
| Solomon Islands | 4882.9 (4223.2-5667.3) | 694.6 (610.5-791.7) | 0.0102 (0.0096-0.0107) |  | 8688.2 (7481.7-10121.7) | 1227.8 (1080.5-1404.1) | 0.0101 (0.0096-0.0106) |  | 295.3 (191.6-429) | 41.6 (27.4-60.1) | 0.0099 (0.0071-0.0128) |
| Somalia | 190391 (161141.8-225074.2) | 800.3 (698.9-916) | 0.0094 (0.0083-0.0105) |  | 340211.9 (287513.3-403698.8) | 1412.2 (1240.7-1602.6) | 0.009 (0.0079-0.0101) |  | 11488 (7434.5-16787.4) | 47.7 (31.3-68.4) | 0.0233 (0.0193-0.0273) |
| South Africa | 467678.3 (408109.3-534662.5) | 849.4 (743.7-971) | -0.0114 (-0.0139--0.009) |  | 823921.3 (720731.6-939430.3) | 1499.2 (1309.1-1711.3) | -0.0112 (-0.0136--0.0088) |  | 27989.2 (18152.9-40382.8) | 50.8 (33-73.6) | -0.0136 (-0.0156--0.0115) |
| South Sudan | 84682.3 (71981.2-99801.1) | 799.8 (698.3-916.1) | 0.0304 (0.0262-0.0347) |  | 150939.4 (128222.4-177655.5) | 1411.5 (1240-1606) | 0.0303 (0.0261-0.0344) |  | 5084.5 (3310.3-7502.7) | 47.5 (31.2-68.4) | 0.0372 (0.0318-0.0426) |
| Spain | 266500.6 (243937.6-290134) | 532.6 (483.4-583.2) | -0.0219 (-0.0285--0.0153) |  | 469363 (429802-511640.8) | 947.6 (864.1-1038.9) | -0.0213 (-0.0279--0.0147) |  | 15544 (10357.4-21883.9) | 31.6 (20.8-44.5) | -0.0225 (-0.0297--0.0154) |
| Sri Lanka | 161385.8 (142946.8-181265.3) | 749.1 (661-845.3) | -0.0099 (-0.011--0.0087) |  | 283557.9 (250532.4-318219.6) | 1321.2 (1159.7-1491.6) | -0.0092 (-0.0103--0.0082) |  | 9646.1 (6424.2-13584) | 44.9 (29.9-63.5) | -0.0037 (-0.0066--9e-04) |
| Sudan | 405314.5 (344099.1-472577.7) | 915.1 (798.9-1045.6) | -0.0061 (-0.0071--0.0051) |  | 717408 (613302.8-832093.1) | 1614.3 (1415.1-1842.5) | -0.0053 (-0.0063--0.0042) |  | 24471.8 (15961.2-35827.9) | 54.8 (35.9-78.7) | -5e-04 (-0.0035-0.0026) |
| Suriname | 4458.4 (3912.3-5058.7) | 800.5 (698.9-917.2) | 2e-04 (-0.0012-0.0017) |  | 7829.6 (6900.6-8872.8) | 1412.7 (1241-1604.6) | 4e-04 (-0.001-0.0019) |  | 266.8 (173.7-386.6) | 48 (31.3-69.7) | -0.0048 (-0.0087--9e-04) |
| Sweden | 84802.9 (75559.3-94480.7) | 800.9 (706.8-898.7) | -0.0132 (-0.0148--0.0116) |  | 149051.5 (132758.6-166772.9) | 1417.5 (1253.4-1590.2) | -0.0129 (-0.0145--0.0113) |  | 4977.3 (3273.1-7024.1) | 47.6 (31.1-67.6) | -0.0092 (-0.0116--0.0069) |
| Switzerland | 52632.1 (46763.2-58620.3) | 575.3 (506.8-645.8) | -0.0101 (-0.0166--0.0036) |  | 92493.4 (82409.1-102863.3) | 1018.1 (901.1-1139.1) | -0.0096 (-0.0162--0.0031) |  | 3082.5 (2037.1-4332.5) | 34.2 (22.4-48) | 0.0028 (-0.0033-0.0089) |
| Syrian Arab Republic | 130324 (113605.7-149271.9) | 922.6 (805.5-1054) | 0.0299 (0.0177-0.0422) |  | 228658.4 (199645.8-261524.2) | 1627.3 (1428-1859.2) | 0.0292 (0.0173-0.0411) |  | 7801.2 (5056.6-11257.4) | 55.2 (35.8-79.1) | 0.0216 (0.0141-0.0291) |
| Taiwan (Province of China) | 149134.3 (131717.2-166454.6) | 704.5 (616.5-800.2) | 0.0149 (0.014-0.0158) |  | 261345.7 (232168.7-291431.6) | 1245.3 (1088.7-1423.9) | 0.0151 (0.0143-0.0159) |  | 8896 (5864.1-12621.8) | 42.6 (27.9-61.1) | 0.0191 (0.0158-0.0224) |
| Tajikistan | 112332.5 (95757.9-130828.7) | 1093 (944.9-1250.2) | -0.0111 (-0.013--0.0091) |  | 198533 (170025.4-231283.1) | 1925.1 (1671.7-2208.4) | -0.0109 (-0.0128--0.009) |  | 6827 (4401.5-9998) | 66 (42.7-95.5) | -9e-04 (-0.0042-0.0025) |
| Thailand | 466220.1 (409855.5-523753.6) | 718 (627.5-819.2) | 5e-04 (0-9e-04) |  | 818087.3 (723848.5-919416.7) | 1270.4 (1113.7-1443.3) | 6e-04 (1e-04-0.001) |  | 27781.1 (18334.3-39073.4) | 43.2 (28.5-61.4) | 0.0181 (0.0144-0.0217) |
| Timor-Leste | 10243 (8772.8-11868.6) | 719.1 (628.4-820.6) | 5e-04 (-0.001-0.0019) |  | 18202.6 (15699.2-21170.7) | 1272.2 (1115.1-1444.9) | 5e-04 (-0.001-0.002) |  | 618.2 (402.6-885.6) | 43.1 (28.3-61.3) | 0.0324 (0.0171-0.0476) |
| Togo | 69964.5 (59926.9-81937) | 803.7 (701.9-920.6) | -0.0012 (-0.0016--8e-04) |  | 123884.2 (106284.1-144684.2) | 1418.3 (1247.4-1612) | -9e-04 (-0.0013--5e-04) |  | 4208.7 (2721.6-6138.6) | 48 (31.3-69.3) | 0.0111 (0.0073-0.0149) |
| Tokelau | 10 (8.8-11.5) | 693.8 (609.7-790.6) | -0.0188 (-0.0224--0.0151) |  | 17.8 (15.6-20.5) | 1226.4 (1079.3-1402.4) | -0.0187 (-0.0222--0.0151) |  | 0.6 (0.4-0.9) | 41.7 (27.1-59.8) | -0.0178 (-0.0219--0.0137) |
| Tonga | 743.1 (646-856.1) | 696.3 (612.2-793.6) | 0.006 (0.0041-0.0078) |  | 1314.3 (1141.8-1518.9) | 1230.8 (1083.2-1406.6) | 0.0058 (0.0041-0.0075) |  | 44.8 (29.1-65.5) | 41.8 (27.2-60.4) | 0.0063 (0.0031-0.0095) |
| Trinidad and Tobago | 10167.7 (9020.3-11423.7) | 799 (697.5-915.4) | -0.0052 (-0.0055--0.005) |  | 17822.1 (15798.7-20043.6) | 1409.9 (1238.3-1601.1) | -0.0052 (-0.0055--0.005) |  | 605.6 (395.5-862.9) | 48 (31.4-69.4) | -0.0046 (-0.0082--9e-04) |
| Tunisia | 101363.1 (89099.4-114976.1) | 916.7 (800.8-1046.6) | 0.0031 (0.0021-0.0042) |  | 178023.7 (157166.3-201950.7) | 1617 (1419.5-1845.4) | 0.0031 (0.0021-0.0041) |  | 6084.3 (4004.8-8747.6) | 55.2 (36.1-79.6) | -9e-04 (-0.0042-0.0024) |
| Turkey | 643682.1 (567395.1-725276.9) | 852.6 (748.6-969.6) | -0.0029 (-0.0048--0.001) |  | 1133620.2 (1002018.2-1275612.2) | 1509.4 (1326.8-1711.4) | -0.0029 (-0.0046--0.0011) |  | 38426.8 (25054.6-54761.7) | 51.2 (33.5-73.8) | -6e-04 (-0.0044-0.0032) |
| Turkmenistan | 56636.5 (48780.2-65191.3) | 1090 (942.1-1247.3) | -0.0331 (-0.0335--0.0326) |  | 99834 (86013.4-114887.7) | 1919.6 (1667.3-2199.6) | -0.033 (-0.0334--0.0325) |  | 3429.9 (2214.8-5011.2) | 65.8 (42.6-96.3) | -0.027 (-0.0291--0.0248) |
| Tuvalu | 82.8 (72.4-94.9) | 693.8 (609.8-790.8) | -0.0217 (-0.023--0.0204) |  | 146 (128.2-167.3) | 1226.6 (1079.2-1401.6) | -0.0208 (-0.022--0.0195) |  | 5 (3.3-7.2) | 41.6 (27.4-60) | -0.0225 (-0.0246--0.0204) |
| Uganda | 382860.4 (324235-452866.7) | 803.6 (701.7-920.8) | 0.0038 (0.0018-0.0058) |  | 681560.8 (576176-806420.6) | 1418.1 (1246-1610.9) | 0.004 (0.002-0.0059) |  | 23180.5 (15101.6-34040.9) | 48.1 (31.7-69.7) | 0.0478 (0.0439-0.0517) |
| Ukraine | 431449.3 (383229.7-482919.6) | 1166.8 (1010.8-1341.2) | -0.0073 (-0.0083--0.0064) |  | 754464.6 (672375.6-843465.9) | 2055.1 (1780.2-2371.7) | -0.0073 (-0.0082--0.0064) |  | 25653.8 (16768.9-36798.4) | 70.6 (46-102.7) | 0.0029 (0-0.0058) |
| United Arab Emirates | 70769.6 (61280.9-82237.8) | 880.6 (768.7-1005.5) | -0.0288 (-0.0454--0.0121) |  | 123669.3 (107401.1-142747.8) | 1554.1 (1357.1-1778.2) | -0.0283 (-0.0443--0.0124) |  | 4244 (2745.8-6068.4) | 53 (34.7-76) | -0.0277 (-0.0463--0.0091) |
| United Kingdom | 444185.9 (394019-494832.5) | 648.9 (573.2-727) | -0.012 (-0.0146--0.0093) |  | 781577.1 (698727.7-872033.8) | 1148.6 (1014.9-1287.3) | -0.0116 (-0.0143--0.0089) |  | 25950.2 (17332.9-36404.7) | 38.4 (25.2-54) | -0.0073 (-0.0096--0.0051) |
| United Republic of Tanzania | 518583.1 (440974.2-610269.2) | 803.5 (701.4-920.8) | 2e-04 (-7e-04-0.0012) |  | 922924.8 (785224.8-1085915.5) | 1417.9 (1245.7-1611.6) | 2e-04 (-7e-04-0.0011) |  | 31308.5 (20275.4-46402.4) | 48 (31.4-70.1) | 0.0394 (0.0335-0.0453) |
| United States of America | 2609718.5 (2480340.4-2746051.8) | 892.3 (844.1-946.3) | 0.0088 (-0.0036-0.0212) |  | 4599617.8 (4340327.8-4879705.4) | 1581.1 (1479-1690.3) | 0.0124 (-0.0014-0.0262) |  | 153479.1 (102752.9-213697) | 53.3 (35.5-74.9) | 0.0032 (-0.0114-0.0178) |
| United States Virgin Islands | 760.5 (675.9-853.5) | 803.1 (701.3-920.1) | -0.0032 (-0.0049--0.0015) |  | 1335.5 (1188.1-1493.8) | 1417.1 (1245.2-1610.1) | -0.003 (-0.0047--0.0014) |  | 45.2 (29.7-65.5) | 48.3 (31.5-70.1) | -0.0079 (-0.0116--0.0043) |
| Uruguay | 24429.1 (21596.7-27423) | 764.2 (668.7-869.2) | 0.0044 (0.0035-0.0053) |  | 42892.5 (38137-47985.4) | 1348 (1187.9-1534) | 0.0042 (0.0033-0.0051) |  | 1455 (957.9-2081.5) | 45.9 (30.2-66.2) | 0.0042 (0.0016-0.0067) |
| Uzbekistan | 379359.6 (326381-436901.5) | 1097.1 (948.9-1255.1) | -0.004 (-0.0046--0.0033) |  | 667210.2 (573307-770180.4) | 1932.2 (1678.1-2216.5) | -0.004 (-0.0046--0.0034) |  | 22942.7 (14986.3-33319) | 66.1 (43.4-95.7) | 0.0015 (-0.0017-0.0047) |
| Vanuatu | 2175.5 (1883-2514.3) | 695.1 (610.9-792.2) | 0.0069 (0.0063-0.0074) |  | 3854.1 (3337.7-4471.9) | 1228.5 (1081.2-1405.4) | 0.0069 (0.0064-0.0074) |  | 131.2 (85.5-190.6) | 41.6 (27.2-59.6) | 0.0051 (0.0019-0.0083) |
| Venezuela (Bolivarian Republic of) | 217273.9 (190998.6-246562.9) | 801.2 (699.5-917.9) | -0.0032 (-0.0053--0.0011) |  | 382447.7 (337548.3-432659.6) | 1413.8 (1242-1606) | -0.003 (-0.0051--0.001) |  | 13046.7 (8502-18902) | 48.2 (31.6-69.5) | 9e-04 (-0.0014-0.0032) |
| Viet Nam | 671006.1 (584889-761309.6) | 718.7 (628.1-819.9) | 0.0115 (0.0109-0.0122) |  | 1181133.5 (1036010.6-1337681) | 1271.6 (1114.8-1444.6) | 0.0115 (0.0108-0.0122) |  | 40450.4 (26294.6-57441.8) | 43.4 (28.4-62) | 0.0317 (0.0272-0.0363) |
| Yemen | 318238.1 (269882-372969.4) | 916.8 (801-1046.7) | 0.0044 (0.003-0.0058) |  | 562846 (480550.5-656110.9) | 1617.3 (1418.9-1846.8) | 0.0041 (0.0027-0.0055) |  | 19071.6 (12482.8-28132.7) | 54.5 (35.9-79) | 0.0031 (-8e-04-0.0069) |
| Zambia | 164924.3 (140303.4-194176.9) | 801.6 (699.7-918.7) | 5e-04 (3e-04-8e-04) |  | 292768.4 (249587.4-344242) | 1414.6 (1242.3-1607.5) | 6e-04 (3e-04-9e-04) |  | 9928.3 (6394-14694.4) | 47.8 (31.2-69.5) | 0.0134 (0.0084-0.0184) |
| Zimbabwe | 133025.7 (113812.6-156081.3) | 805.9 (703.7-923.1) | 0.013 (0.0116-0.0143) |  | 235482.9 (202107-275088.6) | 1422 (1249.6-1615.4) | 0.0129 (0.0116-0.0142) |  | 8048.1 (5248.8-11667.7) | 48.3 (31.8-69.5) | 0.0125 (0.0077-0.0174) |
