# Supplementary material for: Burden of and Trends in Urticaria Globally, Regionally, and Nationally from 1990 to 2019: Systematic Analysis
Source: JMIR Public Health Surveill. 2023 Oct 26;9:e50114. doi: 10.2196/50114 (PMC10636626; doi:10.2196/50114)
Supplement: Multimedia Appendix 2 [file publichealth_v9i1e50114_app2.docx]

Supplementary figures

**Figure S1 Global prevalence cases (A), DALYs (C), and incidence cases (E) and Prevalence rate (B), DALYs rate (D), and incidence rate (F) due to urticaria by age.**

**Figure S2 Both (A), female (B), and male (C) DALYs rate and Both (D), female (E), and male (F) DALYs due to urticaria by age in 21 disease burden regions in 2019**

**Figure S3 Both (A), female (B), and male (C) prevalence rate and Both (D), female (E), and male (F) prevalence cases due to urticaria by age in 21 disease burden regions in 2019**

**Figure S4 Both (A), female (B), and male (C) incidence rate and Both (D), female (E), and male (F) incidence cases due to urticaria by age in 21 disease burden regions in 2019**

**Figure S5 Both (A), female (B), and male (C) DALYs rate and Both (D), female (E), and male (F) DALYs number due to urticaria by age in different SDI areas in 2019**

**Figure S6 Both (A), female (B), and male (C) prevalence rate and Both (D), female (E), and male (F) prevalence cases due to urticaria by age in different SDI areas in 2019**

**Figure S7 Both (A), female (B), and male (C) incidence rate and Both (D), female (E), and male (F) incidence cases due to urticaria by age in different SDI areas in 2019**
